# Economical routes to size-specific assembly of self-closing structures

**DOI:** 10.1126/sciadv.ado5979

**Published:** 2024-07-03

**Authors:** Thomas E. Videbæk, Daichi Hayakawa, Gregory M. Grason, Michael F. Hagan, Seth Fraden, W. Benjamin Rogers

**Affiliations:** ^1^ Martin A. Fisher School of Physics, Brandeis University, Waltham, MA 02453, USA.; ^2^ Department of Polymer Science and Engineering, University of Massachusetts, Amherst, MA 01003, USA.

## Abstract

Programmable self-assembly has seen an explosion in the diversity of synthetic crystalline materials, but developing strategies that target “self-limiting” assemblies has remained a challenge. Among these, self-closing structures, in which the local curvature defines the finite global size, are prone to polymorphism due to thermal bending fluctuations, a problem that worsens with increasing target size. Here, we show that assembly complexity can be used to eliminate this source of polymorphism in the assembly of tubules. Using many distinct components, we prune the local density of off-target geometries, increasing the selectivity of the tubule width and helicity to nearly 100%. We further show that by reducing the design constraints to target either the pitch or the width alone, fewer components are needed to reach complete selectivity. Combining experiments with theory, we reveal an economical limit, which determines the minimum number of components required to create arbitrary assembly sizes with full selectivity.

## Teaser

A scheme for creating complex mixtures of interacting nanoparticles allows for the efficient assembly of self-limited structures.

## INTRODUCTION

The design and control of self-assembly pathways is a promising route for creating complex, functional nanomaterials (*1*). Recent successes in colloidal self-assembly focus primarily on synthesizing spatially unbounded, dense crystalline materials or spatially limited architectures, like clusters, membranes, and filaments, whose dimensions are specified by the building block sizes (*2*–*15*). However, Nature is brimming with examples from a different class of structures that have self-regulated cavity sizes that are much larger than the size of the individual building blocks. These self-limiting cavity sizes are essential to the functionality of various biological devices and materials, such as responsive containers that can selectively package specific genetic material, as in viral capsids (*16*), or photonic nanostructures, like those found in some butterfly wings that produce their structural coloration (*17*). Inspired by these examples, synthetic colloidal assembly has recently taken a large leap forward by developing new colloidal building blocks with directional binding, complex geometries, and specific interactions that can target assembly of similar self-limiting architectures, such as icosahedral shells and cylindrical tubules (*18*–*20*).

**Fig. 1.**
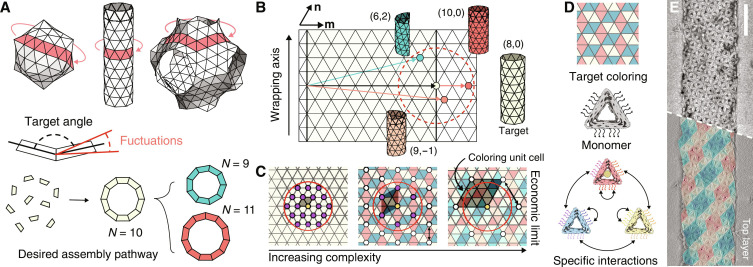
Improving selectivity in self-closing assemblies. (**A**) Examples of self-closing architectures—the unit cell of a Schwarz P-surface (right), a cylinder (middle), and an icosahedral shell (left)—and closed loops of monomers (denoted in red). Fluctuations in the angles between subunits lead to a distribution of closure sizes in loops. (**B**) Schematic of closure fluctuations for a tubule. Tubules form from wrapping a sheet while satisfying a periodic boundary condition. Any discrete assembly can be represented by a unique vector between two vertices on the triangulated plane, corresponding to the circumference of that tubule. The pair of numbers (*m*, *n*) represents the number of steps in the **m** and **n** directions of the lattice to create this circumference vector. States accessible to thermal fluctuations around the target state lie within the dotted circle. Examples of target and off-target assemblies are shown. (**C**) Scheme for removing off-target states. Accessible states are shown as purple, and states allowed by the coloring pattern are white. As the number of colors increases, the distance between similar vertices grows and eventually removes all allowed off-target states from the fluctuation area (red circle). The repeating unit cell of the coloring pattern is shown by the shaded parallelogram. (**D**) Experimental scheme for realizing colorings of tubules. Lines on the monomer edges denote ssDNA locations. Arrows between colored monomers show binding rules. (**E**) Example TEM micrograph for the 16-color tubule. The bottom shows a single layer of the tubule overlaid with the coloring pattern. Ten-nanometer-diameter gold nanoparticles identify the red component. Scale bar, 200 nm.

A common strategy used by Nature to assemble self-limiting structures exploits self-closure (*21*) in which accumulated curvature between bound subunits allows the assembly to close upon itself during growth, terminating assembly in one or more directions of growth. While translating this principle of self-closure could open new doors in synthetic colloidal assembly, self-closure has an associated fundamental challenge that must be solved to assemble precise, self-limiting architectures: Thermal fluctuations give rise to variations in the curvature of the growing assembly, leading to a distribution of the cavity sizes that result in the final structures (*22*). Such variability has been seen with biological subunits, such as the dispersity of the protofilament number of microtubules assembled in vitro (*23*–*25*) and the polymorphism of hepatitis B capsids (*26*), as well as synthetic systems of DNA and de novo proteins that target rings (*18*), tubules (*20*, *27*, *28*), and capsids (*13*, *29*, *30*). This problem relates to the fact that the programmed angles between subunits lead to closed loops around the cavities, as seen for different discrete assemblies in Fig. 1A. Although a certain average curvature may be targeted, the finite bending rigidity of the assemblies leads to a spread of sizes that these loops can form (*21*, *31*–*33*). Moreover, the larger the number of subunits in the loop, the broader the distribution of states that can be accessed, and thus, the ability to selectively assemble the target structure becomes more difficult with increasing loop size. Because the self-limiting cavity sizes are fundamental to how these types of structures function, developing generally applicable solutions to this challenge is essential to realizing synthetic self-limiting architectures with functionalities that rival their natural counterparts. This is particularly critical in the regime of large self-closing sizes where the physical effects of bending fluctuations are increasingly pernicious.

Here, we establish a systematic approach to combat polymorphism by increasing the complexity of an assembly and illuminate the physical relationship that specifies the minimal amount of complexity that needs to be encoded to achieve any single target structure with complete selectivity. Our approach extends symmetry-based theories, such as that from Caspar and Klug (*16*), which identify the minimum number of components required to form a given structure. In particular, we demonstrate our strategy for the specific case of assembling cylindrical tubules from triangular DNA origami subunits (Fig. 1, B to E). We increase the complexity of our assemblies by incorporating a multicomponent, periodic coloring pattern that specifies the arrangement of various components within the tubule. By using colorings with more types of components, fewer tubule geometries are commensurate with both the geometry of the tubule and the periodicity of the coloring, which reduces the number of off-target states that are thermally accessible. By borrowing ideas from two-dimensional (2D) addressable assembly (*13*, *34*, *35*), we decouple the interactions between subunits from the geometry of the monomer to realize assemblies built from up to 16 unique components, demonstrating that our strategy allows assembly with essentially arbitrarily high complexity. Ultimately, we identify an economical threshold—which grows as the size of the cavity squared—where a single assembly state can be selected with a minimal amount of coloring complexity and rationalize this limit using a simple Helfrich energy model. We conclude by showing that one can specifically target lower-dimensional properties, like the width or the pitch, and that this approach provides a more economical scaling that is proportional to the self-limiting size.

## RESULTS

### Improving specificity using multiple components

Our strategy works as follows. A cylindrical tubule can be conceptualized as a sheet that closes upon itself. Because you can tile a sheet with identical equilateral triangles, only one component is required (*20*), and any tubule state can be identified with a unique pair of numbers (*m*, *n*) that corresponds to the shortest closed path around the tubule (directions **m** and **n** in Fig. 1B). But having only a single component also allows the sheet to close on itself in many different ways since all vertices of the triangulation are identical (Fig. 1B). While a single tubule state can be preferred, as specified by the assembly’s curvature, the finite bending rigidity of the sheet could admit many neighboring tubules, with similar curvatures and therefore similar bending energies. These accessible geometries can be thought of as off-target states that occupy an area of vertices around some ground-state vertex. The challenge of eliminating polymorphism in the final structures then boils down to removing all undesired states from within this area.

To accomplish this goal, we color triangles in a periodic way using an increasing number of colors, which removes translational symmetries of the sheet and reduces the number of allowed closure states (Fig. 1C) (*36*). We note that here we are referring to the mathematical concept of coloring and that colors of particles can be implemented through varying the chemical specificity or DNA sequences that mediate interparticle interactions. As additional complexity of coloring is used, the allowed closure states are pushed further apart, and eventually, there are no allowed vertices within the area of fluctuations. At this point, only a single accessible assembly state is permitted by the matching rules.

We develop an experimental system to test this concept, composed of triangular subunits made via DNA origami, that targets a user-specified cylindrical tubule by controlling the interaction specificity and the dihedral angles between neighboring subunits (*20*). To encode the dihedral angles, we bevel each side of the triangle. The monomers also need specific interactions to preserve their orientation with respect to the tubule axis. To realize specific interactions, we place six single-stranded DNA (ssDNA) segments with six-base-long binding domains along each edge (Fig. 1D). Using sticky-end hybridization allows us to encode many low cross-talk interactions (*15*, *35*, *37*–*39*) without changing any internal routing of the DNA origami, which could otherwise have unintended effects on the monomer structure (*19*, *20*, *40*). Last, we assemble tubules at a constant temperature chosen such that the intersubunit interactions are weak and reversible (section S1).

**Fig. 2.**
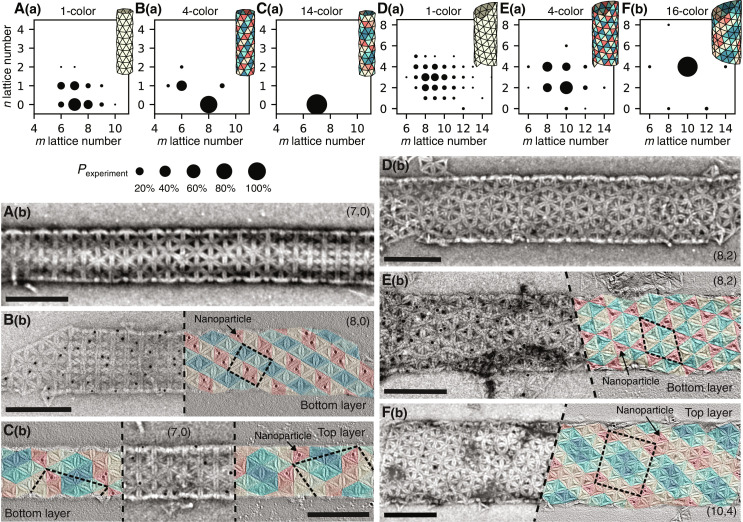
Increased selectivity with increased complexity. (**A** to **C**) (a) Probability distributions of different tubule types for 1-, 4-, and 14-color assemblies with the (6,0) monomer. Each circle denotes a single (*m*, *n*) tubule type, and the area of each point denotes the relative probability. Illustrations of the most probable tubule for each distribution are shown with each plot. Distributions come from measurements of 271, 115, and 153 tubule sections, respectively. (b) Representative TEM images of (6,0) tubule assemblies with the 1-, 4-, and 14-color designs. (**D** to **F**) (a) Probability distributions for 1-, 4-, and 16-color assemblies with the (10,0) monomer. Distributions come from measurements of 241, 271, and 144 tubule sections, respectively. (b) Representative TEM images of (10,0) tubule assemblies with the 1-, 4-, and 16-color designs. Tubules in B(b), C(b), E(b), and F(b) have the corresponding tiling overlaid with a single color (red triangles) labeled with 10-nm-diameter gold nanoparticles. Only positive *n* points are shown since single TEM images cannot differentiate between left and right-handed structures. Scale bars, 200 nm. TEM images have been bandpass-filtered to improve contrast.

We find that a single component assembles a distribution of tubules with varying width and helicity. We first design a triangular monomer that targets a (6,0) tubule and classify the tubules that assemble with transmission electron microscopy (TEM). In particular, we measure both the width and pitch of each tubule to identify its (*m*, *n*) type and then construct a distribution of states (Fig. 2A). The distribution reveals that the monomer prefers to form (7,0) tubules—close to our target of (6,0)—but that over half of the observed tubules form other assembly states distributed near the preferred state, including both achiral and chiral tubules.

To circumvent the formation of off-target states, we engineer the free-energy landscape around the preferred state by building the tubule from a periodic arrangement of a larger number of components. More specifically, we generate a matrix of pairwise subunit interactions by finding periodic colorings of the plane for varying numbers of colors (Fig. 1C and section S2). The set of adjacent colors specifies which unique, specific interactions are required to assemble that pattern, which we implement by designing the sticky-end sequences of our origami subunits. Each color then corresponds to a subunit with a unique set of intersubunit interactions. Going forward, we refer to a component as a “color.” Changing the area and aspect ratio of the unit cell of the periodic colorings allows us to control which tubule states remain accessible to the system.

By choosing colorings that permit the preferred (7,0) state, we aim to reduce the density of nearby, undesired assemblies. Figure 2 (B and C) shows the distributions of 4- and 14-color assemblies. As the number of colors, *N*_colors_, increases, we find that the density of available states decreases, and the probability of the most likely tubule state, which we call “selectivity,” increases. Images of individual tubules show that the quality of assemblies does not diminish as *N*_colors_ increases [Fig. 2, B(b) and C(b)]. We also confirm the specificity of the intersubunit interactions by labeling a single color with 10-nm-diameter gold nanoparticles and performing TEM tomography. While we cannot unambiguously infer the colors of all of the components, we can conclude that the pattern of gold nanoparticles is consistent with our designed coloring in all cases.

Because fluctuations of the dihedral angles between adjacent subunits give rise to the breadth of states, we expect that targeting a larger diameter tubule will result in a broader distribution of off-target structures and thus will require a greater degree of complexity to achieve full selectivity. To test this hypothesis, we design a second DNA origami monomer with bevel angles targeting a (10,0) tubule. Figure 2D shows the distribution of states for this monomer and an example of an assembled tubule. As anticipated, the number of off-target states increases in comparison to the (6,0) tubule. Again, we can improve selectivity through increased assembly complexity. Figure 2 (E and F) shows 4- and 16-color assemblies and a corresponding increase in the assembly selectivity. As before, we see that the quality of tubules is preserved with increasing complexity and that the interaction specificity is consistent with our designed colorings, as seen in the TEM images.

We use a simple Helfrich model to relate the distribution of available states to the physical properties of the assembly (*32*, *41*, *42*). The model assumes that tubule assembly begins with the formation of a circular patch whose curvature can fluctuate. The sheet then grows isotropically until it becomes large enough that it can close upon itself to become a tubule. We assume that once a tubule has closed, it can no longer open and, therefore, has a fixed (*m*, *n*) type. Thus, the distribution of states is determined by the thermal fluctuations at the point of closure. We find that the area of states in (*m*, *n*) space that can form around the target due to thermal fluctuations is proportional to *C*^2^/*B*, where *C* is the preferred circumference nondimensionalized by the monomer edge length and *B* is the bending rigidity nondimensionalized by k_B_*T* (section S7). This area is independent of the assembly complexity. Our coloring patterns introduce another length scale: the distance between similar vertices. This length grows with the size of the unit cell of the coloring. The area of the unit cell increases as *N*_colors_ (Fig. 1C). When these two areas are comparable, we expect that the nearest allowed off-target assembly is outside the area accessible by thermal fluctuations. Thus, we expect a crossover to 100% selectivity when

$$N_{\text{colors}}B/C^{2}≳1$$ (1)

**Fig. 3.**
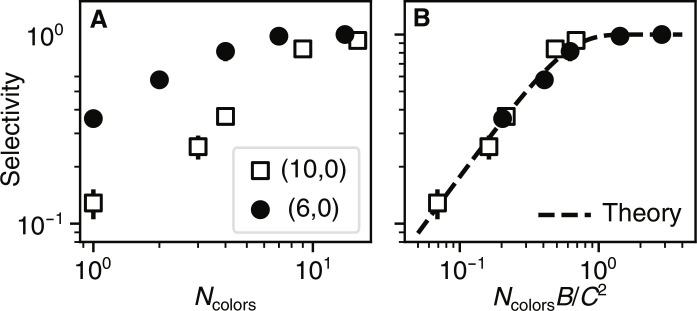
Scaling for increased selectivity. (**A**) Probability of the most common assembly state, called selectivity, against *N*_colors_. (**B**) Selectivity rescaled by the fluctuation area *N*_colors_*B*/*C*^2^. *C* values are taken from the most probable tubule state. Fitting the single-component assemblies to the Helfrich model provides estimated values of the bending rigidity of 10.0 k_B_*T* and 6.7 k_B_*T* for the (6,0) and (10,0) monomers, respectively. See section S7 and (*24*) for a description of the theory.

Our data of the selectivity collapse when replotted according to our Helfrich model. In particular, Fig. 3 shows that the selectivity increases linearly with an increasing number of colors and plateaus to full selectivity when *N*_colors_*B*/*C*^2^ ≈ 1. This crossover point corresponds to the most economical way to target a single assembly state, after which additional complexity provides no further benefit.

### Limiting lower-dimensional properties of assemblies

**Fig. 4.**
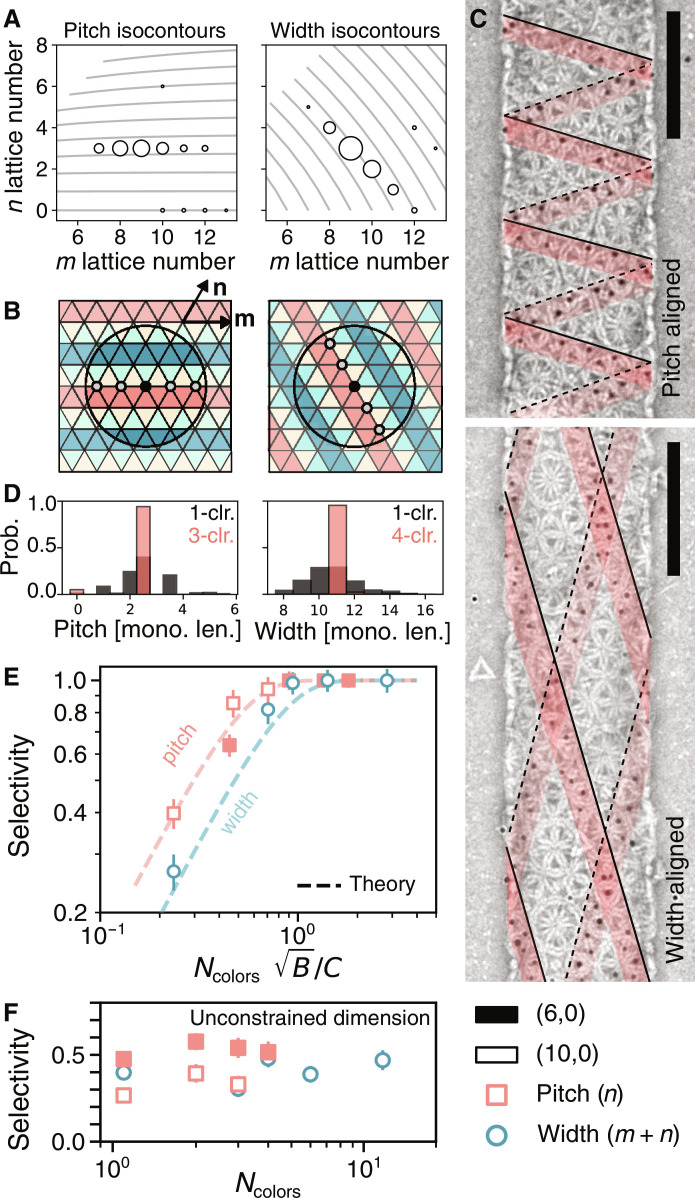
Limiting single degrees of freedom in assembly. (**A**) Isocontours of the pitch and width for tubules for varying *m* and *n*. Experimental distributions overlaid on the plots show that accessible states are lines of neighboring points that lie on lines of constant *n* and *m* + *n*, respectively. (**B**) Schematic of allowed states for linear colorings for pitch or width alignment and (**C**) TEM images of four-color width- and pitch-controlled tubules with the (10,0) monomer. Lines show seams of labeled particles (solid and dashed lines denote the top and bottom layers of the tubule). (**D**) Probability distributions for the pitch and width of multicomponent tubules compared to one-color tubules. Units for the pitch and width are the edge length of a monomer. Complete (*m*, *n*) distributions of these assemblies are shown in (A). (**E**) Selectivity of tubules that have the same *m* + *n* (width-control, circles) or the same *n* (pitch-control, squares). The dashed lines are theoretical predictions from fig. S7. (**F**) Selectivity of the unconstrained dimension versus *N*_colors_.

While the promise of complete selectively is tantalizing, the scaling of the number of components with the square of the self-limited length becomes daunting for larger structures. Therefore, we explore the possibility that targeting a lower-dimensional quantity, like the width or pitch, could be accomplished using fewer components. For each tubule, i.e., each (*m*, *n*), there is a unique width and pitch of the assembly that is dictated by the geometry. We can approximate the width, *w*, and pitch, *p*, of a tubule as *w* = $\sqrt{m^{2}+n^{2}+\mathrm{mn}}$ and *p* = *w* $\sqrt{3}$*n*/(2 *m* + *n*). From these expressions, we can plot lines of constant *w* and *p* on our (*m*, *n*) plot, shown in Fig. 4A. We note that lines of constant *n* or *m* + *n* approximate the lines of constant pitch or width, respectively, particularly in the limit of *m* ≫ *n*, *p =* $\sqrt{3}n/2$ , and in the limit of *m ~ n*, *w =* $\sqrt{3}\left(m+n\right)/2$ . Therefore, we are able to target families of nearly constant width or pitch using linear colorings, in which seams of the same color lie along a single lattice direction, by aligning them along either **n** or **m**-**n** directions (Fig. 4B).

We validate this scheme in experiment and confirm that it yields tubule distributions that are highly selective in their width or pitch. By labeling a single color with gold nanoparticles, we can see that lines of the same color correspond to lattice directions that are closest to the circumferential or axial directions for the pitch- and width-controlled tubules, respectively (Fig. 4C). We also compare the distributions of tubule widths and pitch for two of our (10,0) tubule experiments against a single-color experiment and find that our linear colorings create assemblies with tightly peaked distributions for the desired property (Fig. 4D and fig. S20).

Using a similar argument from the Helfrich model, we find that placing reduced constraints on the assembly leads to an economical threshold that scales linearly with the self-limited length scale rather than quadratically as before. Since we target seams of similar states, we are now concerned not with the area of thermal fluctuations but with their linear extent, *C*/$\sqrt{B}$ . For linear colorings, the separation between the same component type grows as *N*_colors_.

To compare our experimental data to the Helfrich model, we plot the selectivity of tubule states with the same *n* or *m* + *n* against *N*_colors_$\sqrt{B}$/*C*. Figure 4E shows that our data collapse in agreement with the Helfrich model, with the economical point occurring when *N*_colors_$\sqrt{B}/C\approx 1$ . However, we find that reducing the design constraints comes at the cost that the unconstrained dimension (e.g., the pitch when one targets the width) does not see any improvement in selectivity for increasing complexity (Fig. 4F).

**Fig. 5.**
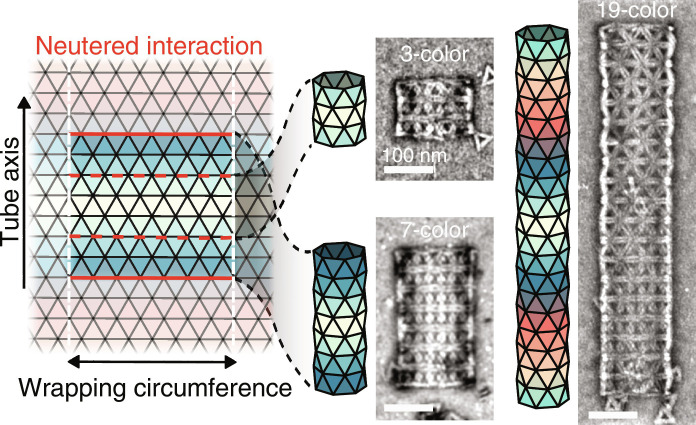
Tubule length limitation. For achiral tubules, such as those made by the (6,0) monomer, a linear coloring constrains tubules to length *n* by neutering one edge of the *n*th color. Dashed and solid red lines denote the neutered interactions for *n* = 3 and 7. TEM images for 3-, 7-, and 19-color assemblies are shown. Scale bars, 100 nm.

Whereas we have so far focused on constraining the (*m*, *n*) states that can form, we conclude by showing how assembly complexity can also be used to constrain a different aspect of the tubule geometry: their length. We use the same class of linear colorings as above, but now we passivate a single edge of one color to prevent the further addition of monomers along that seam (Fig. 5). In this way, the number of colors constrains the tubule length. In Fig. 5, we show representative TEM micrographs of our (6,0) monomer with 3-, 7-, and 19-color assemblies. In all cases, we find that the system reaches an equilibrium state characterized by a mixture of monomers, small clusters, and finite, *n*-length assemblies (section S8). The spirit of this type of constraint is different from those mentioned previously since we are not increasing the complexity to reduce the number of states accessible by thermal fluctuations but rather to cap the growth of assemblies, highlighting the various ways that assembly complexity can be used to engineer self-limitation.

## DISCUSSION

In this work, we have demonstrated a scheme for preventing off-target assemblies by increasing the complexity of the initial assembly mixture. By imposing complex colorings, implemented through matrices of specific intersubunit attractions, assembly states that were accessible due to fluctuations in the curvature become disallowed, leading to full selectivity of the target geometry. By imposing different numbers of constraints on the assembly, we were able to either achieve full selectivity for both the pitch and width of tubules (Figs. 2 and 3) or either one separately (Fig. 4). The benefit of selecting a single dimension of the structure, say the width, is that it can be done using substantially fewer colors, which becomes essential as the self-limiting dimension becomes arbitrarily large relative to the subunit size. Unfortunately, targeting the pitch or width alone comes with a lack of selectivity for the unconstrained dimension, e.g., the pitch. We show examples of this trade-off in fig. S10. While in the limit of large target sizes selectivity eventually falls off, we showed that increasing the complexity mitigates the marginal losses of selectivity, enhanced by a factor of *N*_colors_ over the standard Helfrich expectation.

We anticipate that our strategy is generalizable to other self-closing architectures. A useful point of comparison is the Caspar-Klug (CK) construction for icosahedral capsids (*16*). By using the symmetry of icosahedral shells, the CK rules provide a method for determining the minimal number of components needed to form a shell of arbitrary size. However, for too low of a bending modulus, capsids can assemble asymmetric, defective structures. These defects often come from disclinations that form at fivefold or threefold points. By increasing the number of colors around certain symmetry points in the structure, beyond the minimum prescribed by CK theory, we anticipate that strategies like the one we present here could reduce the propensity for these types of systems to form defects. Examples of such a scheme are illustrated in section S11 for both an icosahedral capsid (*19*) and the triply periodic Schwarz P-surface (*43*). While the benefits of adding complexity by lowering symmetry appear similar at face value to the tubule case in terms of preventing nearby off-target states, there are open questions for these distinct topologies, most notably, what is the density of the nearest off-target states and how do the energy gaps between these off-target states depend on the self-closing size, as well as the relative cost for angular versus stretching distortions of the assembly.

Despite the great improvement in specificity that we see using multicolor assemblies, there are still challenges that need to be overcome. The foremost is the kinetics of growth. The timescale for assembly increases with increasing complexity since the chance of the correct color monomer finding a binding site at the growth front decreases with *N*_colors_ (section S9). This decrease in assembly rate could be compensated for by increasing the monomer concentration, but this strategy could become costly. Instead, future strategies may exploit hierarchical pathways to assembly both for accelerating assembly and possibly reducing the desired complexity if certain kinetic paths can be found that disallow off-target structures. An important aspect of the kinetics that this work reveals is that assembly near the economical point is favorable. In the 14-color assembly with the (6,0) monomer, although all closed structures formed the same (*m*, *n*) state, a small fraction of assemblies missed the point of closure and overgrew as a sheet that wrapped around itself like a scroll (fig. S19). We hypothesize that this type of misassembly will become even more prevalent if the assembly complexity passes the point of optimal economy, *N*_colors_*B*/*C*^2^ ≫ 1.

An important consideration with all types of multicomponent assemblies is the possibility of cross-talk between different components that can stabilize unintended contacts (*34*, *44*). In this context, we can ask why our designs worked so well and do not seem to suffer from the inevitability of cross-talk between a limited set of DNA sequences (*38*)? Here, we highlight that the site addressability of DNA origami allows for the inclusion of interaction geometry as an additional knob for tuning the specificity of interactions (*35*). For some of our interaction patterns, we reused strands from other interactions but altered their arrangement on the faces of the interacting edges. This combination of sequence specificity and spatial commensurability greatly increases the number of unique interactions that can be specified by a finite library of DNA sequences. Furthermore, increasing the number of sticky ends per side could be used to increase this combinatoric aspect further, allowing for many more components than have been explored here.

Coloring, the process of finding allowed, distinguishable identities for particles in a structure is an attractive way to increase the complexity of assemblies, although the inverse design challenge of finding an appropriate coloring for a desired outcome is not always straightforward. Here, we were able to exploit the symmetries and periodicity of the tubule to generate any desired coloring. More complex geometries, such as capsids or gyroids, require considering local point symmetries of the structure (*16*, *19*, *43*, *45*) to encode the correct interactions, while fully addressable clusters often reveal their allowed colorings through detailed searches of all possible interactions (*46*). Recent efforts have also shown that colorings can be used to encode hierarchical structures into crystalline self-assemblies (*15*). As assembly structures become more complex, finding the right scheme to encode large libraries of interactions may require new methods, such as SAT-assembly (*47*, *48*). In addition, it will be equally important to be able to predict and thus program the right binding free energies to facilitate robust assembly (*49*–*51*).

Another successful DNA nanotechnology platform for assembling tubules uses small complexes of DNA, called DNA tiles, as building blocks (*27*, *52*). However, this system has a couple of key differences from our origami-based approach that fundamentally alter the mechanism of self-closure and the design principles for precisely targeting tubules of a specific diameter and helicity. First, owing to the high bending flexibility of the DNA tiles, self-closure is kinetically driven, and tubule assembly is biased toward the narrowest tubules that can form. As a consequence, precisely specifying the tubule diameter using multicomponent assembly requires going all the way to the fully addressable limit (*53*), in which the self-limiting diameter is controlled directly by the number of components, in contrast to our approach, in which the economical limit is determined by a balance between geometrical specificity and interaction specificity. Second, the rigidity of the DNA tiles is anisotropic, resulting in a wide dispersity in the diameters but a narrow dispersity in the helicity of assemblies (*54*). Therefore, tile assembly only requires using multiple “colors” of tiles to program the diameter, whereas our origami approach requires using coloring to control both the diameter and the pitch. Beyond coloring, other strategies have also been used to program the assembly of tubules from DNA tiles, such as using well-defined seeds to template growth (*28*) or exploiting steric interactions between bound nanoparticles (*55*). It will be exciting to see how these types of strategies can be translated to cases like ours that use subunits with vastly different mechanical properties.

Going forward, our scheme could be expanded to systems with subunits with both unique interactions and unique geometries to target a wider range of self-limiting architectures. Whereas we focused on a base monomer with fixed geometry, other target structures have the need for varying subunit geometries, as with icosahedral shells (*19*) or for surfaces with varying Gaussian curvature, such as helical cylinders, toroids, or open crystalline structures (*43*). Moreover, many studies have recently demonstrated that DNA origami can be dynamically reconfigured (*56*–*59*), so it is within sight to imagine a base monomer with adjustable edge lengths or bevel angles that could be used to produce new nanoscale devices.

## MATERIALS AND METHODS

### Folding DNA origami

To assemble our DNA origami monomers, we make a solution with 50 nM p8064 scaffold (Tilibit), 200 nM each staple strand [Integrated DNA Technologies (IDT); nanobase structures 234 and 235 (*60*) for sequences], and 1× folding buffer. We then anneal this solution using a temperature protocol described below. Our folding buffer, from here on referred to as FoB*X*, contains 5 mM tris base, 1 mM EDTA, 5 mM NaCl, and *X* mM MgCl_2_. We use a Tetrad (Bio-Rad) thermocycler to anneal our samples.

To find the best folding conditions for each sample, we follow a standard screening procedure to search multiple MgCl_2_ concentrations and temperature ranges (*20*, *40*) and select the protocol that optimizes the yield of monomers while limiting the number of aggregates that form. All particles used in this study were folded at 17.5 mM MgCl_2_ with the following annealing protocol: (i) hold the sample at 65°C for 15 min, (ii) ramp the temperature from 58° to 50°C with steps of 1°C/hour, and (iii) hold at 50°C until the sample can be removed for further processing.

### Agarose gel electrophoresis

We use agarose gel electrophoresis to assess the folding protocols and purify our samples with gel extraction. We prepare all gels by bringing a solution of 1.5% (w/w) agarose in 0.5× tris-borate-EDTA (TBE) to a boil in a microwave. Once the solution is homogeneous, we cool it to 60°C using a water bath. We then add MgCl_2_ and SYBR-safe (Invitrogen) to have concentrations of 5.5 mM MgCl_2_ and 0.5× SYBR-safe. We pour the solution into an Owl B2 gel cast and add gel combs (20-μl wells for screening folding conditions or 200-μl wells for gel extraction), which cools to room temperature. A buffer solution of 0.5× TBE and 5.5 mM MgCl_2_, chilled at 4°C for an hour, is poured into the gel box. Agarose gel electrophoresis is run at 110 V for 1.5 to 2 hours in a 4°C cold room. We scan the gel with a Typhoon FLA 9500 laser scanner (GE Healthcare) at 100-μm resolution.

### Sample purification

After folding, we purify our DNA origami particles to remove all excess staples and misfolded aggregates using gel purification. If the particles have self-complementary interactions, then they are diluted 2:1 with 1× FoB2 and held at 47°C for 30 min to unbind higher-order assemblies. The folded particles are run through an agarose gel (now at a 1× SYBR-safe concentration for visualization) using a custom gel comb, which can hold around 2 ml of solution per gel. We use a blue fluorescent light table to identify the gel band containing the monomers. The monomer band is then extracted using a razor blade. We place the gel slices into a Freeze ‘N Squeeze spin column (Bio-Rad), freeze it in a −20°C freezer for 5 min, and then spin the solution down for 5 min at 12 krcf. The concentration of the DNA origami particles in the subnatant is measured using a NanoDrop (Thermo Fisher Scientific). We assume that the solution consists only of monomers, where each monomer has 8064 base pairs.

Since the concentration of particles obtained after gel purification is typically not high enough for assembly, we concentrate the solution using ultrafiltration (*40*). First, a 0.5-ml Amicon 100-kDa ultrafiltration spin column (Millipore) is equilibrated by centrifuging down 0.5 ml of 1× FoB5 buffer at 5 krcf for 7 min. Then, the DNA origami solution is added and centrifuged at 14 krcf for 15 min. We remove the flow-through and repeat the process until all of the DNA origami solution is filtered. Last, we flip the filter upside down into a new Amicon tube and spin down the solution at 1 krcf for 2 min. The concentration of the final DNA origami solution is then measured using a NanoDrop.

### Tubule assembly

Assembly experiments are conducted with DNA origami particle concentrations ranging from 2 to 30 nM. For assemblies that are made up of multiple colors, the quoted concentration is the total concentration of all subunits, e.g., for a 10-nM experiment with *N* colors, each color has a concentration of 10/*N* nM. Assembly solutions have volumes up to 50 μl with the desired DNA origami concentration in a 1× FoB20 buffer. The solution is placed in a 200-μl polymerase chain reaction (PCR) tube and loaded into a thermocycler (Bio-Rad), which is held at a constant temperature, ranging between 30° and 50°C. The thermocycler lid is held at 100°C to prevent condensation of water on the cap of the PCR tube.

Since DNA hybridization is highly sensitive to temperature, we expect that there should be a narrow range of temperatures over which the system can assemble by monomer addition. To make sure that we assemble tubules within this regime, we prepare many samples over a broad range of temperatures. At high temperatures, we find that there are no large assemblies, implying that we are above the melting transition for our ssDNA interactions. As we lower the temperature, we find a transition to the formation of assembled tubules but with increasing defect density as the temperature decreases. For this reason, all assembly experiments are conducted just below the melting transition.

### Labeling tubules with gold nanoparticles

We first attach thiol-modified ssDNA (5′-HS-C_6_H_12_-TTTTTAACC-ATTCTCTTCCT-3′, IDT) to 10-nm-diameter gold nanoparticles (AuNP) (Ted Pella) using a protocol similar to that in (*61*). We first reduce the thiolated strands using tris(2-carboxyethyl) phosphine (TCEP) solution (Sigma-Aldrich) by holding a mixture of 10 mM TCEP (pH 8) and 100 μM thiol-DNA at room temperature for 1 hour on a vortex shaker. We remove excess TCEP with a 10-kDa Amicon filter in three washes of a 50 mM Hepes buffer (pH 7.4); we follow this with filter centrifugation at 4 krcf for 50 min at 4°C. After purification, we store thiolated DNA strands at −20°C until needed. To attach thiolated DNA to AuNPs, we mix DNA with AuNPs at a ratio of 300:1 in a 1× borate buffer (Thermo Fisher Scientific) and rotate the mixture at room temperature for 2 hours. After incubation, we increase the salt concentration in a stepwise manner to 0.25 M NaCl using a 2.5 M NaCl solution in five steps. After each salt addition, we rotate the AuNP solution at room temperature for 30 min. After the last addition, we let the AuNP solution age in the rotator overnight. To remove excess thiol-DNA strands, we wash the DNA-AuNP conjugates four times by centrifugation using a 1× borate buffer with 0.1 M NaCl. In each wash step, we centrifuge the DNA-AuNP solutions at 6.6 krcf for 1 hour. After the last wash, we measure the DNA-AuNP concentration using a NanoDrop and store the solution at 4°C.

To attach AuNPs to tubules, we incorporate handles on the interior edges of the DNA origami subunit with a complementary sequence (5′-AGGAAGAGAATGGTT-3′, IDT) to the DNA on the AuNP. For a multicomponent assembly, only one subunit type has handles that bind to the AuNPs. After tubules have been assembled, we dilute the assembly solution into a mixture with final concentrations of 1 nM DNA origami monomers and 2 nM AuNP in 1× FoB20 and incubate at 32°C overnight. After incubation, samples are ready to be prepared for imaging.

### Fluorescence microscopy

We incubate our DNA origami tubules with YOYO-1 dye (Invitrogen) at room temperature for a minimum of half an hour in a solution of 5 nM monomers, 500 nM YOYO-1, and 1× FoB at the MgCl_2_ concentration of the assembly. This ratio of YOYO-1 to DNA origami is chosen so that there are 100 dye molecules per structure, a limit in which the dye’s impact on the structural integrity of the origami should be negligible (*62*). A total of 1.6 μl of the solution is pipetted onto a microscope slide that has been cleaned with Alconox, ethanol (90%), acetone, and deionized water and subsequently plasma-cleaned. After deposition, a plasma-cleaned coverslip is placed on the droplet at an angle and carefully lowered so that the liquid film is as thin as possible. We find that this reduces the sample thickness to about the width of a tubule without damaging the tubules, allowing them to lie flat on the surface. Samples are imaged on a TE2000 Nikon inverted microscope with a Blackfly USB3 (FLIR) camera.

### Negative-stain TEM

We first prepare a solution of uranyl formate (UFo). We boil doubly distilled water to deoxygenate it and then mix in UFo powder to create a 2% (w/w) UFo solution. We cover the solution with aluminum foil to avoid light exposure and vortex it vigorously for 20 min, after which we filter the solution with a 0.2-μm filter. Last, we divide the solution into 0.2-ml aliquots, which are stored in a −80°C freezer until further use.

Before each negative-stain TEM experiment, we take a 0.2-ml UFo aliquot out from the freezer to thaw at room temperature. We add 4 μl of 1 M NaOH and vortex the solution vigorously for 15 s. The solution is centrifuged at 4°C and 16 krcf for 8 min. We extract 170 μl of the supernatant for staining and discard the rest.

The electron microscopy (EM) samples are prepared using FCF400-Cu grids (Electron Microscopy Sciences). We glow discharge the grid before use at −20 mA for 30 s at 0.1 mbar, using a Quorum Emitech K100X glow discharger. We place 4 μl of the sample on the carbon side of the grid for 1 min to allow adsorption of the sample to the grid. During this time, 5- and 18-μl droplets of UFo solution are placed on a piece of parafilm. After the adsorption period, the remaining sample solution is blotted on 11-μm Whatman filter paper. We then touch the carbon side of the grid to the 5-μl drop and blot it away immediately to wash away any buffer solution from the grid. This step is followed by picking up the 18-μl UFo drop onto the carbon side of the grid and letting it rest for 30 s to deposit the stain. The UFo solution is then blotted, and any excess fluid is vacuumed away. Grids are allowed to dry for a minimum of 15 min before insertion into the TEM.

We image the grids using an FEI Morgagni TEM operated at 80 kV with a Nanosprint5 complementary metal-oxide semiconductor camera (AMT). The microscope is operated at 80 kV, and images are acquired between ×8000 and ×20,000 magnification.

### TEM tomography

To obtain a tilt series, we use an FEI F20 equipped with a Gatan Ultrascan 4k × 4k charge-coupled device camera, operated at 200 kV. The grid is observed at ×18,000 magnification from −50° to 50° in 2° increments. The data are analyzed, and the *z*-stack is reconstructed using IMOD (*63*).

### Cryo–electron microscopy

Higher concentrations of DNA origami are used for cryo-EM grids than for assembly experiments. To ensure that particles remain isolated from each other in the ice, we use passivated monomers, which have no ssDNA strands protruding from the faces of the DNA origami. To prepare samples, we fold between 1 and 2 ml of the folding mixture (50 nM scaffold concentration), gel purify it, and concentrate the sample by ultrafiltration, as described above. EM samples are prepared on glow-discharged C-flat 1.2/1.3 400 mesh grids (Protochip). Plunge-freezing of grids in liquid ethane is performed with an FEI Vitrobot with sample volumes of 3 μl, blot times of 16 s, a blot force of −1, and a drain time of 0 s at 20°C and 100% humidity.

Cryo-EM images for the (10,0) DNA origami monomer are acquired with the FEI Tundra TEM with a field emission gun electron source operated at 100 kV and equipped with an FEI Falcon II direct electron detector at a magnification of ×59,000. Single-particle acquisition is performed with SerialEM. The defocus is varied from −0.5 to −4 μm with a pixel size of 2.023 Å.

Cryo-EM images for the (6,0) DNA origami monomer are acquired with a Tecnai F30 TEM with a field emission gun electron source operated at 300 kV and equipped with an FEI Falcon II direct electron detector at a magnification of ×39,000. Single-particle acquisition is performed with SerialEM. The defocus is set to −2 μm for all acquisitions with a pixel size of 2.87 Å.

### Single-particle reconstruction

Image processing is performed using RELION-3 (*64*). Contrast transfer function estimation is performed using CTFFIND4.1 (*65*). After picking single particles, we perform a reference-free 2D classification from which the best 2D class averages are selected for processing, estimated by visual inspection. The particles in these 2D class averages are used to calculate an initial 3D model. A single round of 3D classification is used to remove heterogeneous monomers, and the remaining particles are used for 3D autorefinement and postprocessing. Figures S21 and S22 show views of the reconstructions and the resolution curves. The postprocessed maps are deposited in the Electron Microscopy Data Bank with entries EMD-43226 and EMD-43227.

## Supplementary Materials

### This PDF file includes:

Sections S1 to S11 Figs. S1 to S23 Tables S1 to S5 References
