# Supplementary material for: Economical routes to size-specific assembly of self-closing structures
Source: Sci Adv. 2024 Jul 3;10(27):eado5979. doi: 10.1126/sciadv.ado5979 (PMC11221488; doi:10.1126/sciadv.ado5979)
Supplement: Supplementary file 1 — Sections S1 to S11 Figs. S1 to S23 Tables S1 to S5 References [file sciadv.ado5979_sm.pdf]

Science Advances

Supplementary Materials for

Economical routes to size-specific assembly of self-closing structures

Thomas E. Videbæk *et al.*

Corresponding author: Thomas E. Videbæk, videbaek@brandeis.edu; W. Benjamin Rogers, wrogers@brandeis.edu

Sci. Adv. **10**, eado5979 (2024)
DOI: 10.1126/sciadv.ado5979

This PDF file includes:

Sections S1 to S11
Figs. S1 to S23
Tables S1 to S5
References

S1. DETERMINING ASSEMBLY CONDITIONS FOR SSDNA INTERACTIONS

Since DNA hybridization is sensitive to both salt species and concentration as well as the temperature of assembly we conduct a screening of a variety of conditions to see where we find tubule assembly. To explore this we conducted assembly experiments with both $MgCl_2$ (Fig. S1A) and NaCl (Fig. S1B) as the cation. For both of these salts, we looked for assemblies at different temperatures. In general, regardless of the cation species or concentration, there is a characteristic temperature, called the melting temperature, that separates the solution having assemblies and the solution remaining as monomers. We have seen that the closer an experiment is to the melting temperature the better the overall quality of tubules that we find, see the TEM images in Fig. S1A. We did not find an appreciable difference in the quality of the tubule between the types of salts. At too high of a salt concentration, about 30 mM $MgCl_2$, the melting temperature begins to approach the disassembly temperature of our DNA origami subunits. For all of our experiments we choose to use a concentration of 20 mM $MgCl_2$ where we saw a melting temperature of about 36 °C.

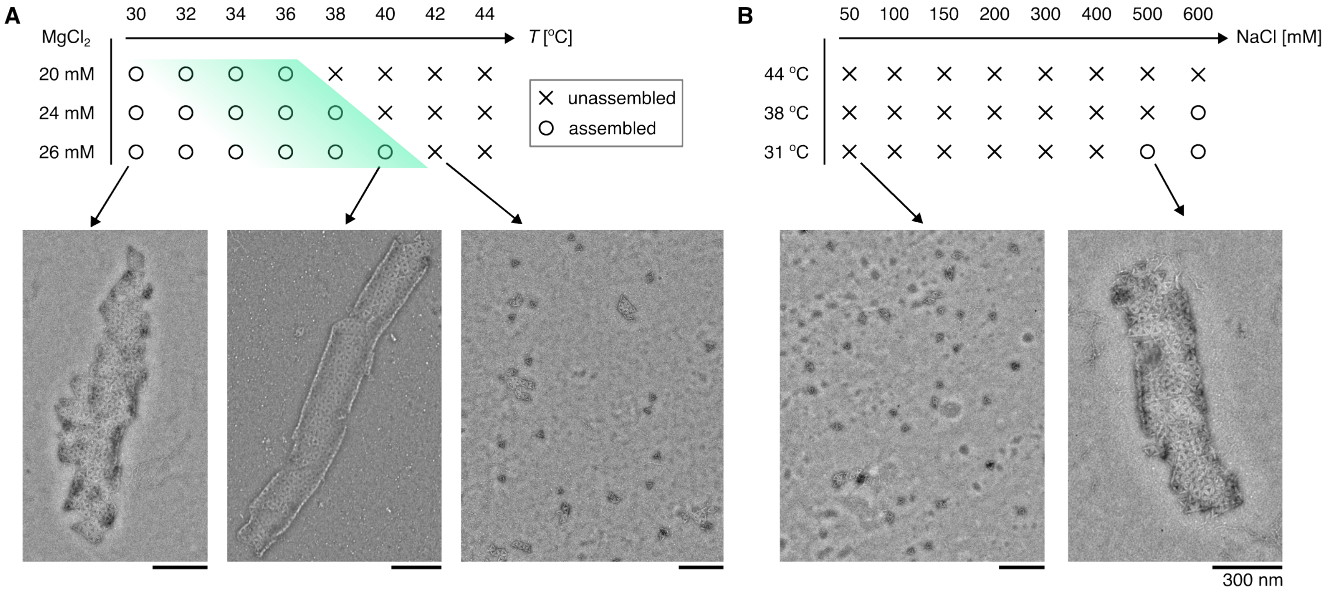

FIG. S1. **Assembly conditions for tubule assembly.** (**A**) Phase diagram for assembly with $MgCl_2$ concentration and temperature. The gradient over the assembly symbols represents a decrease in assembly quality at lower temperatures. (**B**) Phase diagram for assembly with NaCl concentration and temperature. Selected images show representative assemblies at a few points in the diagrams. All TEM images have been bandpass filtered.

S2. GENERATING COLORING PATTERNS FOR TUBULES

To know what interactions to program between our monomer species, we first need to generate coloring patterns. A coloring pattern is the assignment of color to each triangle on the plane such that it can be wrapped into a tubule. This restriction means that the coloring needs to have translational symmetry and can have at most 2-fold symmetry since tubules have a well-defined axis (these criteria correspond to either 'o' or '2222' symmetry groups on the plane). To minimize the number of colors needed to create a tubule, we only use patterns with 2-fold symmetry in this work. Our previous study [32] used a brute-force computational search over all possible interactions between subunits to generate allowed patterns, but this strategy becomes intractable for more than ten colors.

Here we generate coloring patterns by prescribing a desired unit cell for the tiling and then using symmetry to color in the pattern. In Fig. S2, we outline the basic procedure. First, we choose a primitive cell for the coloring, which is a parallelogram that can tile the plane by translation and is defined by two primitive vectors for its side length. Next, we assign a color to an arbitrary triangle that lies within the parallelogram. Since we require that our parallelogram has both translational symmetry and 2-fold rotational symmetry, some triangles within the parallelogram are required to have the same color to preserve symmetry. After assigning a color, we color in any symmetric triangles in the pattern. We continue this method of coloring until all the triangles in the parallelogram have been assigned a color. A benefit of this type of pattern generation is that it is simple to create a multi-color assembly that targets a specific tubule state. Since the primitive vectors of the unit cell define the periodicity of the coloring, they also inform one of the tubule states that will be geometrically commensurate with that coloring. A more detailed description of this scheme can be found in ref. [36].

S3. DESIGN OF INTERACTION SEQUENCES

To have all of the required interactions for our multi-component assemblies, we need to have a library of unique interactions that have low crosstalk. We use an algorithm described by Nadrian Seeman [37] to construct our library. A sketch of the algorithm is as follows. First, we choose a set of bases (letters) that we will use to construct our sequences, such as AGT for three letters. Using these letters, we create a list of all their permutations of up to some length N. We call this set our dictionary. After populating the dictionary with all permutations, we remove all entries that contain the same letter at least three times in a row, such as AAA. This dictionary is then used to generate a new sequence (word) of some target length M, where M is greater than N. To create a word, (i) we pick an entry at random from our dictionary and set it to be the start of the word, (ii) we remove the entry from the dictionary as well as its complement, if it is there, (iii) we pick a new entry from our dictionary that starts with the last N-1 letters of our current word and add the last letter of our picked entry to the word, (iv) we remove the picked entry and its complement from the dictionary, (v) we repeat steps (iii) and (iv) until the word reaches the target length M, and (vi) we repeat steps (i-v) until the dictionary is depleted to the point that new M-letter words cannot be formed.

Using a four-letter alphabet (ACGT) with five-letter dictionary entries, we generated a list of 187 six-letter words. From this list, we choose 72 words such that they, and their compliments, had the lowest off-target interactions. These sequences are listed in Table S1 along with their calculated binding free energy to their complements at 1 M NaCl [66].

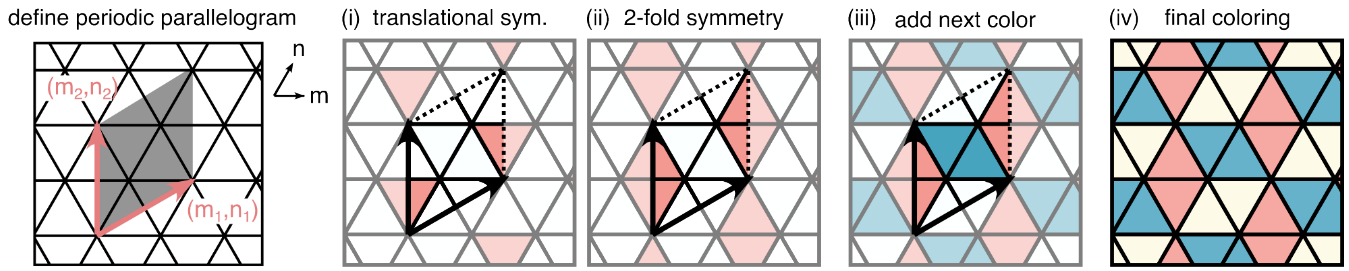

FIG. S2. **Generation of colorings.** Outline for coloring generation scheme. First, choose a parallelogram that will tile the plane, defined by primitive vectors (m_1, n_1) and (m_2, n_2). To color in the parallelogram, and subsequently the plane, we use the following steps: (i) assign a color to an arbitrary triangle in the parallelogram and color any other triangles that can be reached by any of the primitive vectors, (ii) color any triangles that can be reached by a colored triangle from a 2-fold rotation about the center of the parallelogram, (iii) repeat the previous steps for an uncolored triangle with a new color until (iv) all triangles in the parallelogram have a color assigned.

S4. LIBRARY OF SIDE INTERACTIONS

Given a list of sequences with minimal crosstalk, we also need to generate a library of interactions for each side of each subunit species. For any multi-component design, there can be both self-complementary and non-self-complementary interactions. For each triangular subunit, we want six strands on each of its three sides. Given this design criteria, we create a list of multiple self-complementary and non-self-complementary sequences for each side. These strands are listed in Table S2 along with the sum of their binding free energies. The set of strands for each side was chosen so that they would have similar total binding free energies to each other. Due to the limited size of our interaction sequence library, some sets of six strands share the same sequence. In these cases, the locations of the sequences were chosen to minimize off-target binding. Estimates of binding energies are shown in Fig. S3.

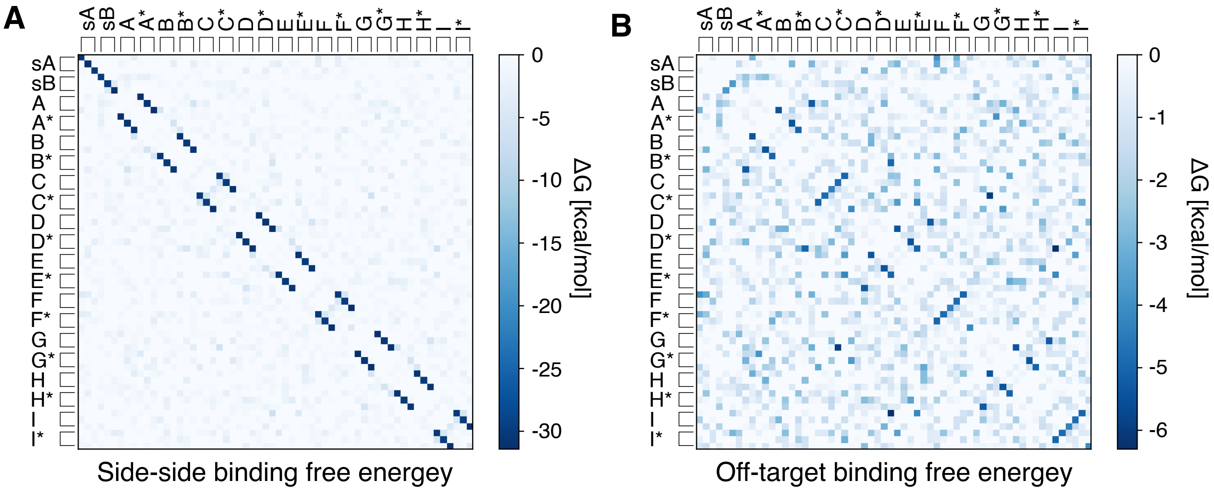

FIG. S3. **Side-side interaction energy.** (**A**) Free energy of binding between different sets of interactions is shown in Table S2. Each set of three rows and columns (denoted by the bracket) corresponds to the side 1, 2, 3 sequences in that order. (**B**) To highlight the weaker binding energy of the off-target interactions, we set all intended binding interaction energies to 0 kcal/mol and rescaled the colorbar.

S5. ANALYSIS OF TUBULE DISTRIBUTIONS

After assembly experiments, we observe the assembled structures with TEM. We find many tubules that are deposited upon the mesh that present a variety of tubule types. Since our tubules are made of a discrete number of subunits, we know each tubule, or section of a tubule, can be identified with a unique (m, n) pair, where m and n describe the shortest path around the circumference of the tubule while moving allong the $\mathbf{m}$ and $\mathbf{n}$ lattice directions, as shown in Fig. 1B of the main text. As described in the work of Hayakawa et al. [20], each type of tubule can be identified with a specific circumference and maximum seam angle. By measuring each of these quantities for all tubules we image, we can create a distribution of the tubule states that are accessible to the system.

We illustrate this analysis by looking at the data set for the 4-color isotropic case from Fig. 2E in the main text. In Fig. S4A, we show a TEM image of a tubule and the measured seam angle and width in the image. Measuring many tubules allows us to build up a distribution of seam angles and circumferences, Fig. S4B. We assume that the measured width is half the circumference since tubules become flattened during grid prep [20]. Since the tubule does not lie perfectly flat, may crack slightly when deposited on the TEM grid, or have a varying amount of stain, there is some spread in the values away from those expected for discrete tubules. For the case of a multi-component assembly, only some of the states are expected to be allowed, as shown by the red squares. The data cluster about these points, but the spread is comparable to the separation between possible tubule types. Since the AuNP labeling and tomography experiments show high fidelity of interaction specificity, we cluster these points to their nearest allowed state to construct an (m, n) distribution for the assembly, Fig. S4C. The nearest state is the one that minimizes the sum in quadrature of the differences between the experimental and the expected values for discrete, flattened tubules of the seam angle and circumference.

To increase our confidence in the distributions we measure, we perform an additional analysis in which we identify the n number directly from TEM images. In Fig. S4D, we show that the seams of triangles in both layers of the

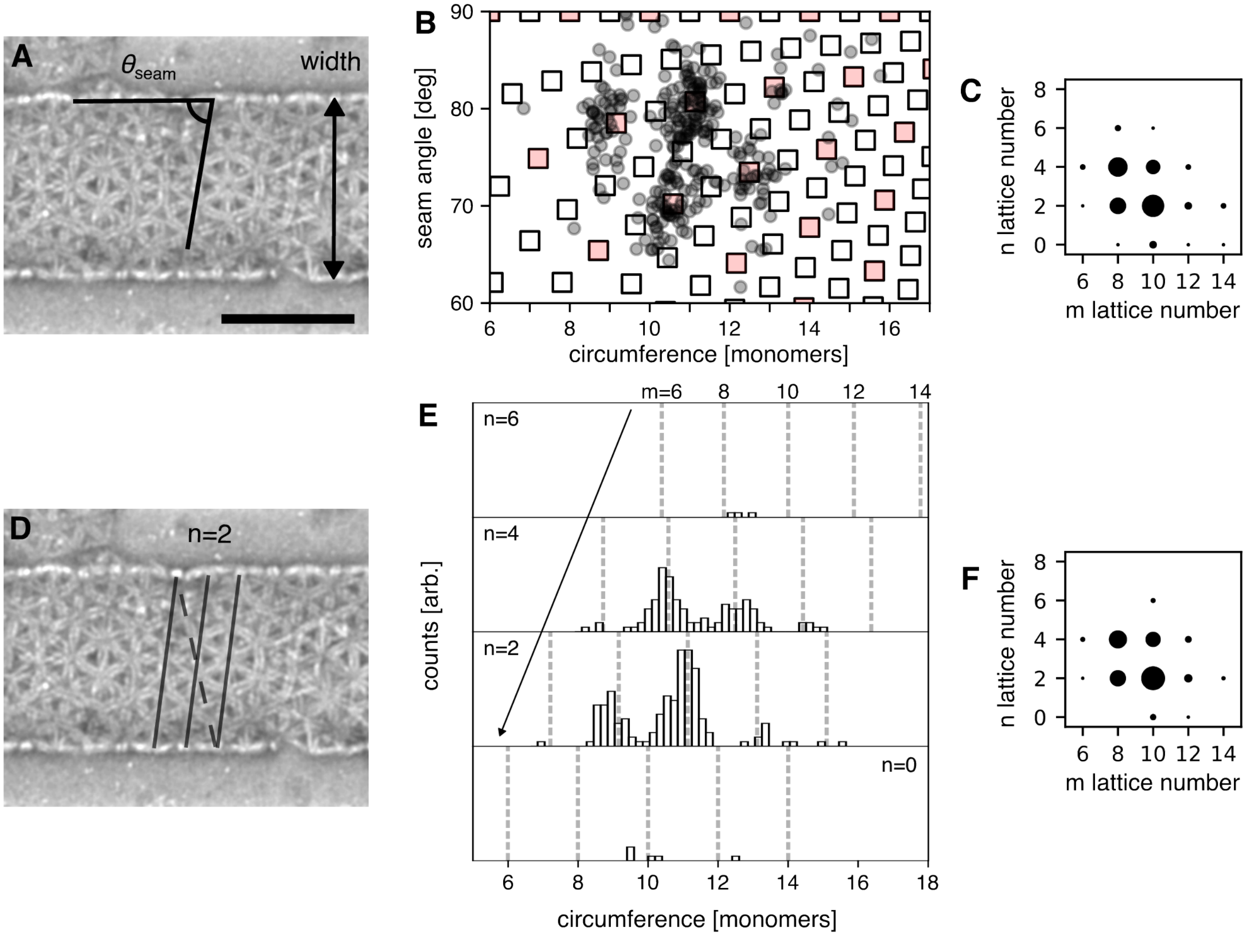

FIG. S4. **TEM tubule analysis** (**A**-**C**) Width and seam angle analysis. (**A**) For any tubule, one can measure the seam angle, θ_{seam}, and the width on the TEM gird. This image is from the 4-color experiment shown in Fig. 2 for the (10,0) monomer in the main text. Scale bar is 200 nm. (**B**) Plot of the seam angle versus the diameter of the tubule. We take the diameter to be twice the measured width. Open square denote all discrete tubule states, filled squares are the states allowed by the coloring of triangles. (**C**) By clustering points to the nearest allowed state from the seam angle versus width data, we construct the (m, n) distribution for the assembly. (**D-F**) Width and n analysis. (**D**) By looking at the lattice seams of the tubule one can directly infer the n state of the tubule. The solid line shown seams for one layer of the tubule and the dashed line is the seam on the other layer. In this image we see that there are exactly two rows of triangles that span the dashed line, so we know this is an $n = 2$ tubule. (**E**) Histograms of the diameters of tubules for each corresponding n number measured in TEM. Dashed vertical lines are the diameter for tubules with m numbers 6 to 14 with the corresponding n number and allowed by the coloring. Note that the histograms show peaks only at the allowed m numbers. (**F**) By clustering points in each histogram to their nearest allowed m number we construct the (m, n) distribution. Images have been bandpass filtered.

tubule can be seen. By matching up where the seams for two layers meet, we can directly infer the n number for each tubule. This procedure is a more reliable method of determining the n number than using the seam angle since those data show a broad spread compared to the separation between discrete tubule states. After determining n, we construct histograms of the measured circumferences for each set of unique n numbers, as shown in Fig. S4E. Looking at the circumferences, we see distinct peaks that match up well with the states that are allowed from the coloring. Again we can cluster these points to the nearest allowed tubule state and construct an (m, n) distribution (Fig. S4F). This method provides a quantitatively similar distribution to the one described above. For all distributions shown in the main text, we use this second method.

We also note that for lower complexity assemblies, tubules have a tendency to change their type along their length, see Fig. S13. When we encounter such tubules we consider each section that has a continuous type as an individual occurrence of closure. In the instance shown in Fig. S13 we would count four separate instances in our data.

S6. ESTIMATING ASSEMBLY YIELD

While we do not see unintended side products of assembly, there are assembled tubules, monomers, and small clusters in solution. In Fig. S16 and S17, we show overview images of TEM grids for tubule assemblies. From these types of images, we can try to estimate the yield of assembly, i.e. the fraction of monomers that fruitfully enter into tubules. To estimate this we measure the areal density of both tubules and small clusters in the TEM images.

To get the areal density of monomers and small clusters, ρ_{free}, we measure the density of origami on sections of the images that are free of tubules. First, we manually count the number of monomers in a large field of view. We then bandpass filter and threshold the same region of monomers to measure the area fraction that they take up in the image. The image processing smears out the monomers and overestimates their area. The manual count is then used to calibrate this over-estimation. We then use this calibration to get the areal density of monomers in several fields of view.

To get the areal density of monomers in tubules, ρ_{tubule}, we measure the area of tubules in a field of view. To do this we approximate each tubule as a rectangle and manually place rectangles over all of the tubules in an image. Since tubules have two layers we multiply the area of all these rectangles by two. The areal density is the ratio of the scaled tubule area to the area of the field of view.

To get the final estimate for the assembly yield we compare the fraction of monomers in tubules to all monomers in a field of view, $\rho_{\text{tubule}}/(\rho_{\text{tubule}} + \rho_{\text{free}})$. For the 1-color assembly shown in Fig. S16 we find that around 75% of monomers are in tubules. For the 9-color assembly shown in Fig. S17 we find that around 60% of monomers are in tubules.

S7. HELFRICH MODEL OF THERMAL FLUCTUATIONS

A. Predicting tubule-type distributions

To understand which neighboring tubule states are within the reach of thermal fluctuations, we consider how fluctuations of curvature for a growing sheet relate to the mechanical properties of the sheet. We use a Helfrich model for the elastic energy of the sheet [22, 41], which goes as $E = \frac{1}{2}BA(\Delta\kappa_\perp)^2$, where A is the area of the assembly, B is the bending rigidity, and $\Delta\kappa_\perp$ is the fluctuation of the curvature in the circumference direction.

We assume that the growth of assemblies in our system roughly follows the path shown in Fig. S5. Subunits form a circular patch, which adopts some amount of curvature that is related to the binding angles programmed into the subunits; this curvature is also subject to thermal fluctuations. If the binding energies for each side of the triangular subunits are similar, we assume that the patch will then grow isotropically until it is large enough to close upon itself. After closure, the assembly is classified as a tubule and can continue growth by extending. From prior work [20, 22, 32], we assume that the assembly of the is slow and monomers attach to and detach from the growing assemblies, so that it can be modeled as quasi-equilibrium. Thus, the curvature and subunits can anneal. In contrast, the tubule type is frozen in when the sheet closes on itself, because at this point disassembly involves breaking multiple bonds and thus a large activation barrier. Hence, the curvature and subunits are quenched after closure. This assumption means that the dispersity of states in the assembly can be directly tied back to the fluctuations of the pre-closure sheet.

From this simple picture of growth, we can estimate the fluctuations of our assembled states by finding the energies of assemblies at the closure point and then infer their distribution assuming that the probabilities follow a Boltzmann distribution. First, we need an estimate for the area of sheets at closure. At the closure point, the diameter of the patch matches the circumference of the tubule it has closed into, $2r_{\text{patch}} = 2\pi R$, where r_{patch} is the radius of the assembled sheet and R is the radius of curvature of the tubule. Using this relation we get that the assembly has an area of $A = \pi^3 R^2$. Second, we need to express the fluctuation of curvatures in terms of the fluctuations of the circumference of the tubule, C. This is given by:

$$(\Delta\kappa_\perp)^2 \approx \frac{1}{R^2}\left(\frac{\Delta C}{C}\right)^2. \tag{2}$$

Combining these two expressions we find that the Helfrich energy for a sheet at the closure point is

$$E_{\text{H}} = \frac{1}{2}\pi^3 B\left(\frac{\Delta C}{C}\right)^2. \tag{3}$$

Assuming that the circumferences of the tubules follow the Boltzmann distribution, $P = \exp(-E_{\text{H}}/k_{\text{B}}T)/Z$, where Z is the partition function, then the standard deviation of the circumference goes as $\Delta C \propto C/\sqrt{B}$.

We extend this Helfrich model to the (m, n) space of tubule types by considering a simple elastic model for the energy of our assembly. Since the triangular monomers have a preferred binding angle on each side, any deviation from this angle will result in an elastic energy cost. For an assembly with N components, the elastic energy can be estimated as

$$E = \frac{1}{4} NB \sum_{i \in 1,2,3} (\theta_i - \theta_{0,i})^2, \tag{4}$$

where $\theta_{0,i}$ is the preferred binding angle and θ_i is the actual binding angle for side i. We can estimate the size of an assembly at closure as we did above, finding $N = (4\pi/\sqrt{3})(C/l_0)^2$, where l_0 is the edge length of a subunit. As a note, we can estimate the difference in binding angles for different size tubules and find that $\Delta\theta \propto \Delta C/C^2$. Putting this result into Eqn. 3 gives the same scaling as in Eqn. 2. Since both N and $\theta_{0,i}$ depend upon m and n, this model gives a state-dependent probability $P(m, n)$ that we also assume follows a Boltzmann distribution.

B. Scaling law for isotropic colorings

Making some simplifying assumptions about this model lets us infer a scaling law for how the increase in selectivity of tubule states depends on the number of colors and properties of the tubule for isotropic colorings. We note that the addition of complexity through coloring does not change the elastic energy of any of the assembly states. Instead, the change in the allowed states causes the remaining states to have increased probability through rescaling the partition function. Essentially, the probability that would have gone into assembly states that become disallowed is funneled into neighboring allowed states. However, since the breadth of the distribution only depends on the mechanics of the assembly, e.g. the bending rigidity, this results in an increase in the probability of making the target state.

To calculate the increase in probability we calculate the cumulative probability of states within an area that depends on the area of the unit cell of the coloring. In Fig. S6A, we show an (m, n) distribution of tubule states generated by Eqn. 3 overlayed on the vertices of the triangular lattice of monomers. The distribution of states has two well-defined axes, one shorter than the other, and the distribution of these states is fairly Gaussian, owing to the θ^2 dependence of the elastic energy. To account for the coloring patterns of states, we note that for isotropic patterns, similar vertices in the coloring are separated by a distance of $\sqrt{N_{\text{colors}}}$. When this coloring pattern is imposed on the tubule some states become inaccessible and their probabilities are funneled into neighboring available states (Fig. S6B). To get an estimate for the increase in selectivity, we integrate the tubule state probability distribution within a circle of radius $\sqrt{N_{\text{colors}}}$, whose origin is the center of the distribution of assembly states. To make this calculation simpler, we assume that the tubule distribution is radially symmetric such that the selectivity takes the form

$$P \propto \int_0^{2\pi} \int_0^{\sqrt{N_{\text{colors}}}} r \exp(-r^2/(C^2/B)) \mathrm{d}r \mathrm{d}\theta, \tag{5}$$

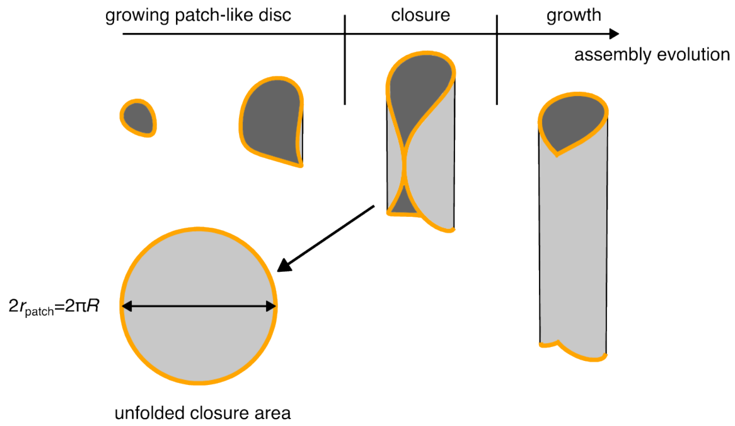

FIG. S5. **Sketch of early assembly growth.** At early times, the assembly will grow as a circular patch a radius of curvature imposed by the subunit binding angles and subject to thermal fluctuations. Once the sheet is large enough, it can close on itself, at this point the curvature can no longer fluctuate. After closure, the assembly is now a tubule and can continue to elongate. The orange color represents the boundary of the sheet.

where the standard deviation of the Gaussian is taken to be $C/\sqrt{B}$. Evaluating this integral gives an approximate equation for the selectivity of

$$P \propto \exp(-N_{\mathrm{colors}} B/C^2). \tag{6}$$

The important takeaway from this result is that the selectivity depends upon $N_{\mathrm{colors}} B/C^2$, which we use to rescale our experimental data in Fig. 3 in the main text. Though we make approximations to make this calculation straightforward, numerical studies of Eqn. 3 find the same scaling for the selectivity [32].

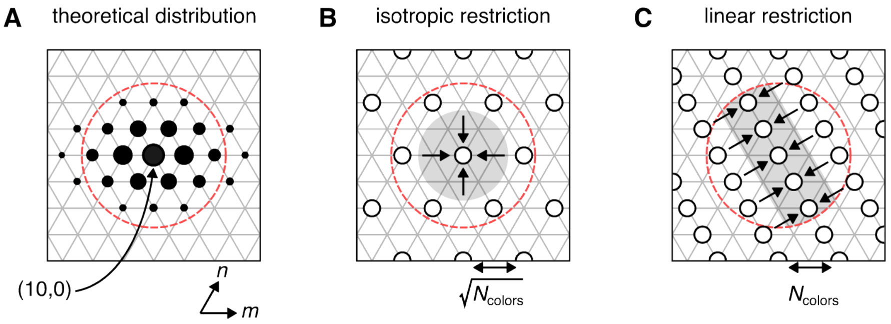

FIG. S6. **Scaling for selectivity with N_{colors}.** (**A**) Tubule distribution generated from Eqn. 3 targeting a (10,0) state with a bending modulus of 8 k_BT. (**B**) By using isotropic colorings we remove possible states from around the target state in an area. The distance between states scales with $\sqrt{N_{\mathrm{colors}}}$. Since some states can no longer close into the tubules due to the coloring pattern, probability from these states gets funneled into neighboring available states. (**C**) With linear colorings the separation between states grows as N_{colors}. White circles on the plane denote similar vertices based on an unspecified coloring.

C. Scaling law for linear colorings

Using the same type of argument outlined above, we can make an estimate for the scaling law for the selectivity of the width- and pitch-control experiments outlined in Fig. 4 in the main text. As shown in Fig. S6C, the linear colorings have states that are funneled to a seam of allowed points. To get an estimate of the scaling for how these colorings change selectivity, we need only integrate over one dimension of the Gaussian distribution. Since the separation between the seams grows as N_{colors}, we have that

$$P \propto \int_{-\infty}^{\infty} \int_{-N_{\mathrm{colors}}}^{N_{\mathrm{colors}}} \exp(-(x^2+y^2)/(C^2/B))\mathrm{d}x\mathrm{d}y, \tag{7}$$

where x and y correspond to a change in basis with x pointed along the seam of similar states and y perpendicular to it. This integration results in

$$P \propto \mathrm{erf}(N_{\mathrm{colors}}\sqrt{B}/C). \tag{8}$$

This equation reveals that the scaling for the selectivity has changed to depend upon $N_{\mathrm{colors}}\sqrt{B}/C$, which we use to rescale the data in Fig. 4E.

D. Comparison with experiment

To compare this model with experiment, we look at distributions of tubules generated by Eqn. 4 and see how the distributions change when different tubule colorings are used to restrict states. For the isotropic colorings, we use the results from Videbæk et al. [32]. For the pitch- and width-controlled cases, we generate distributions for a variety of bending rigidities and preferred tubule geometries using Eqn. 4, and restrict their distributions using linear colorings. Scaling this data with $N_{\mathrm{colors}}\sqrt{B}/C$ collapses the data and shows that the pitch- and width-controlled cases split into two curves, as seen in Fig. S7. These two curves arise due to the slight asymmetry of the tubule distribution in the (m, n) space (Fig. S6A). The dashed lines in Fig. S7 show the same curves as shown in Fig. 4E in the the main text.

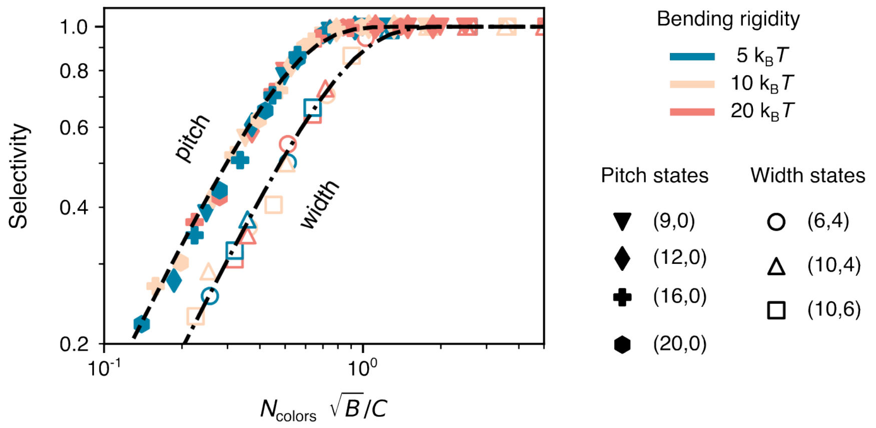

FIG. S7. **Numerical simulations for pitch and width controlled tubules.** Selectivity versus the rescaled linear number of colors for pitch- and width-controlled tubules based on the Helfrich model. Open symbols are width-controlled and full symbols are pitch-controlled. A variety of bending rigidities and tubule circumferences are shown. The dark lines show approximations to the curves and are used in Fig. 4E in the main text.

S8. LENGTH-CONTROLLED TUBULE GEL

Since the length-controlled tubules are finite assemblies, it is possible to observe their evolution toward equilibrium with gel electrophoresis. We prepare assemblies at different points in time and run them in a 0.5% agarose gel to separate different-sized assemblies. Figure S8A shows a representative gel of this type of kinetics experiment. At early times, we see that the assemblies are mostly monomers with some signal coming from larger assemblies, though it is possible that some of this occurs from assembly at low temperatures in the pocket of the gel. At later times, we see the emergence of distinct bands. We infer the geometries of the tubules in these bands by comparing them to the (m, n) distribution of a 2-color pitch-controlled tubule experiment (Fig. S8B). In the tubule distribution, the smallest structure is a (6,0) tubule, the most probable state is (7,0), and there is a tail at larger m. We see a similar structure for the bands in the gel scan and conclude that larger diameter length-controlled tubules travel slower in the gel. We note that we did not characterize the (m, n) distribution from TEM images of length-controlled tubules since the length-controlled tubules often break during deposition on the grid, making the interpretation of widths unreliable for these small structures.

To make sure that our assembly gel is reliable we perform a careful analysis on the intensity of the gel lanes. For each lane, we first correct for the background intensity of the gel by using an empty lane (not shown in the figure). We then normalize each lane by the sum of its intensity, including any signal that comes from the pocket of the gel (Fig. S8C). Looking closely at this signal we see that even though there is a noticeable increase in the assembly peak intensity, there is a small decrease in intensity of the broad peak on the right. This means that the noticeable increase to the peak in the assembly bands is due to a small decrease across the broad distribution of monomers and small oligomers. To confirm this we measure the integrated intensity of the oligomer and assembly bands in the gel (Fig. S8D). Over time the oligomer intensity drops while the assembly intensity increases. The small drop in total intensity of these two regions of the gel is due to some structures remaining in the pocket of the gel.

S9. KINETICS OF TUBULE GROWTH

To see how changing the number of components in an assembly changes the growth rate, we perform kinetics experiments that quantify the length of tubules over time. Since our assemblies grow to several microns in length, it is simple to observe them with epi-fluorescence microscopy. After staining the assemblies with an intercalating dye (YOYO-1), we take images of the tubules at different points in time. Figure S9A shows exemplary images for a 7-color experiment over ten days, with larger structures appearing at later times. To quantify the change in the lengths, we binarize the images, fit the particles to ellipses, and take the fitted major lengths as the lengths of the tubules. By performing this analysis for a set of thirty images, we generate a distribution of lengths for different points in time (Fig. S9B). For each time point, we extract the mean length, L_0, of the distribution. Simple models for the growth of one-dimensional filaments suggest that the relevant parameters for rescaling the assembly time scale are the initial monomer concentration, c, and the number of colors, N_{colors}, since these directly impact the rate of monomer addition

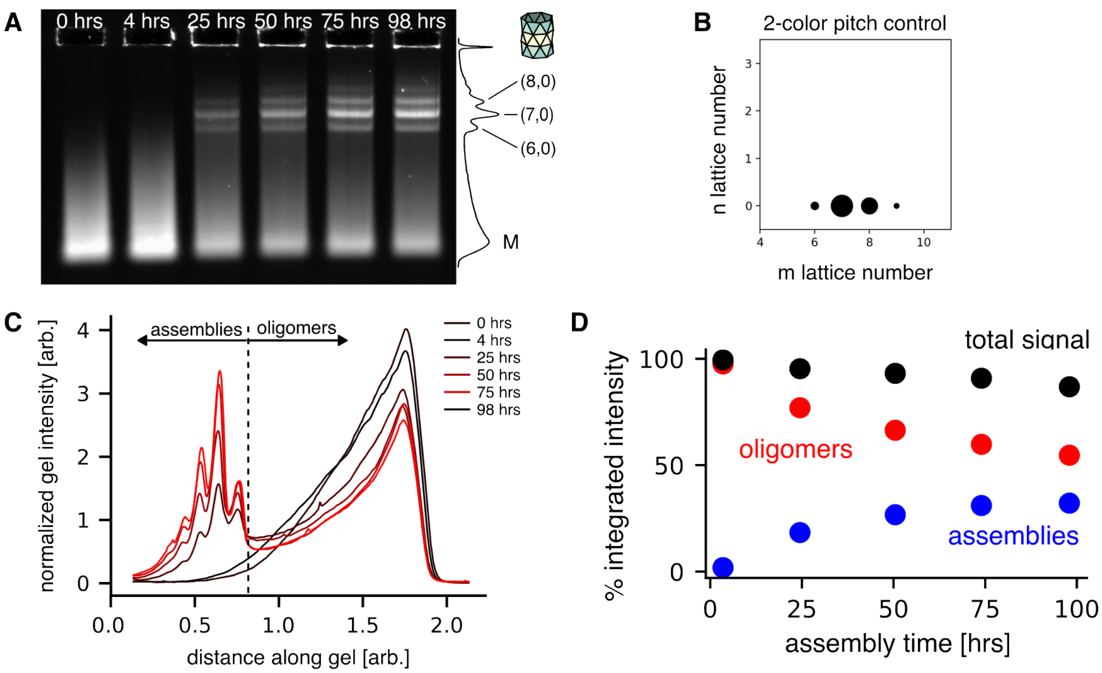

FIG. S8. **Kinetics of length controlled tubules** (**A**) Scan from a gel electrophoresis experiment of the 3-color length controlled tubules of the (6,0) monomer for different assembly times. On the right is a plot of the fluorescence intensity for the 98 hr assembly. M denotes the monomer band. (**B**) Tubule distribution for a 2-color pitch-controlled assembly using the (6,0) monomer. (**C**) Background corrected and normalized fluorescence intensities for the gel lanes shown in (A). (**D**) Plot of the fractional integrated intensity for the oligomers (red) and assembled length-controlled tubules (blue) (signal to the right and left of the dotted line in (C), respectively), plotted against the assembly time. The black points are the sum of these two signals. The roughly ten percent decrease in signal comes from the structures that are trapped in the pocket of the gel.

for a growing assembly [67]. In Fig. S9C, we plot L_0 versus a rescaled time, ct/N_{colors}, and find that our data collapse to a single curve for a range of N_{colors} and initial concentrations.

A. Analysis of fluorescence images for kinetics

Due to the way that we binarize our images, there is a lower bound for the size of objects that we can detect. This lower bound directly manifests in the distributions that we measure. Since we want to measure the average length, L_0, of the tubule distribution, we need to incorporate this lower bound into extracting the correct average length. We implement this correction by integrating over an exponential distribution from this lower bound, L_c, instead of 0,

$$\frac{\langle L^2\rangle}{\langle L\rangle} = \frac{\int_{L_c}^{\infty}(l^2/L_0)\exp(-l/L_0)\mathrm{d}l}{\int_{L_c}^{\infty}(l/L_0)\exp(-l/L_0)\mathrm{d}l} = \frac{2L_0^2 + 2L_0L_c + L_c^2}{(L_c + L_0)}, \tag{9}$$

where L_c is the cutoff length scale. By measuring the experimental value of $\langle L^2\rangle/\langle L\rangle$ and using Eq. 9, we can solve for L_0 of the tubule lengths distribution. For all measured distributions, we use a consistent cut-off of $L_c = 500$ nm.

S10. TRADE-OFFS IN SELECTIVITY

As described in the main text, we introduced a strategy for targeting either the pitch or the width of a tubule to get a more favorable scaling for the complexity by limiting a single dimension of the thermal fluctuations. This allows one to prescribe a specific assembly property with significantly fewer colors than when selecting for a single assembly state. Of course, this comes with the trade-off that the unconstrained dimension gains no benefit in selectivity with increasing complexity. We illustrate this in Fig. S10 by plotting the selectivity of assemblies using either the singly- or doubly-selective strategies. Based on our experimental data, Fig. 3 and 4 of the main text, and numerical calculations, Fig. S7, the distributions of tubules match our Helfrich model. Using this model we plot the theoretical

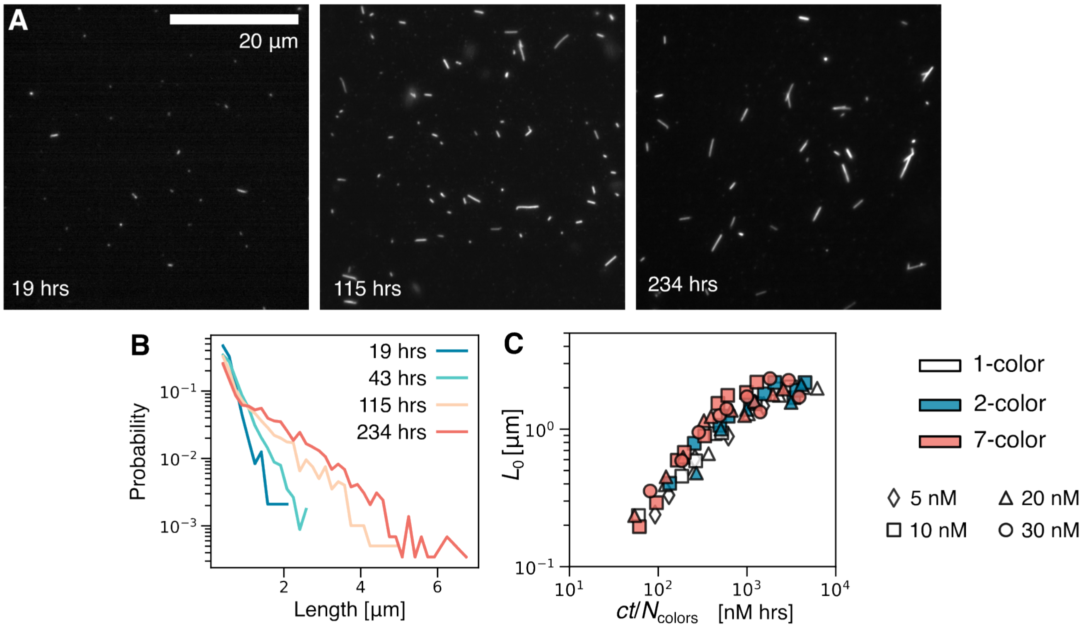

FIG. S9. **Kinetics of multi-color tubule growth** (**A**) Fluorescent microscopy images of a 7-color tubule assembly at different points of time. (**B**) Length distributions of tubules at different points in time. (**C**) Average length of tubules, L_0, plotted against a rescaled time, ct/N_{colors}.

width-selectivity for assemblies with either the singly- or doubly-selective strategies against both the assembly size and the number of colors, Fig. S10A. For another way to view this trade-off, we also plot separately the doubly-selective prediction, Fig. S10B, and singly-selective prediction, Fig. S10C, along with our experimental data. For the singly-selective plots, we show both the pitch- and the width-selectivity. These color maps emphasize the improvement of selectivity for larger self-limiting sizes,

S11. EXTENDING COMPLEX ASSEMBLY TO OTHER TOPOLOGIES

Here we present a direction for how to expand the results we have shown for tubules to other self-closing structures. For the case of tubules, we showed that there is a large degeneracy of states that are isometric to one another, which requires only slight changes in the binding angles. Analogous situations arise in assembling other self-closing topologies, which we illustrate for both an icosahedral capsid and a triply-periodic Schwarz P-surface in Fig. S11. These assemblies can most naturally form defects at symmetry points within the structure (e.g. n-fold rotation axes). While leveraging symmetry is essential for the economy of design, it is also a pathway to off-target states. When trying to assemble structures with a minimal number of components, binding angle fluctuations can cause the formation of shells or cages of different symmetries. While the examples shown in Fig. S11 are of a small size, they represent a large class of structures. As shown in the Caspar-Klug construction for capsids [16] and an analogous one for triply-periodic polyhedra [43]. Larger triangulations with these symmetries can be designed, associated with the so-called T-number, for example by simple subtriangulation of the simplest forms we show. In Fig. S11 we show examples of adding complexity to these types of assemblies by using colorings to reduce the symmetry of the objects. This reduction in symmetry removes off-target states from the energy landscape. How the selectivity of these types of geometries scales with size, bending rigidity, and complexity is an open question that will be the focus of a future study.

The nature of the isometric off-target states for shells and triply-periodic polyhedral is subtly different than for tubules. Whereas the symmetry of the tubule is preserved for all states, off-target states for capsids and triply-periodic surfaces naturally involve a change in symmetry as the unintended states correspond to the insertion or removal of a triangle at symmetry points of the structure. Due to this change, it is unclear how the density of these off-target states changes as we move to larger structures and in particular, how close these off-target states are in terms of their elastic energy. For reasons illustrated above, for tubules, the energy gap (per particle) between a target state and its nearest off-target competitor decreases with size. Removal/addition of finite angular wedges around symmetric vertices required by misassembly of capsids and P-surfaces may lead to substantially larger gaps in energy between target and nearest off-target states and therefore warrant further investigation. In any case, the use of increased

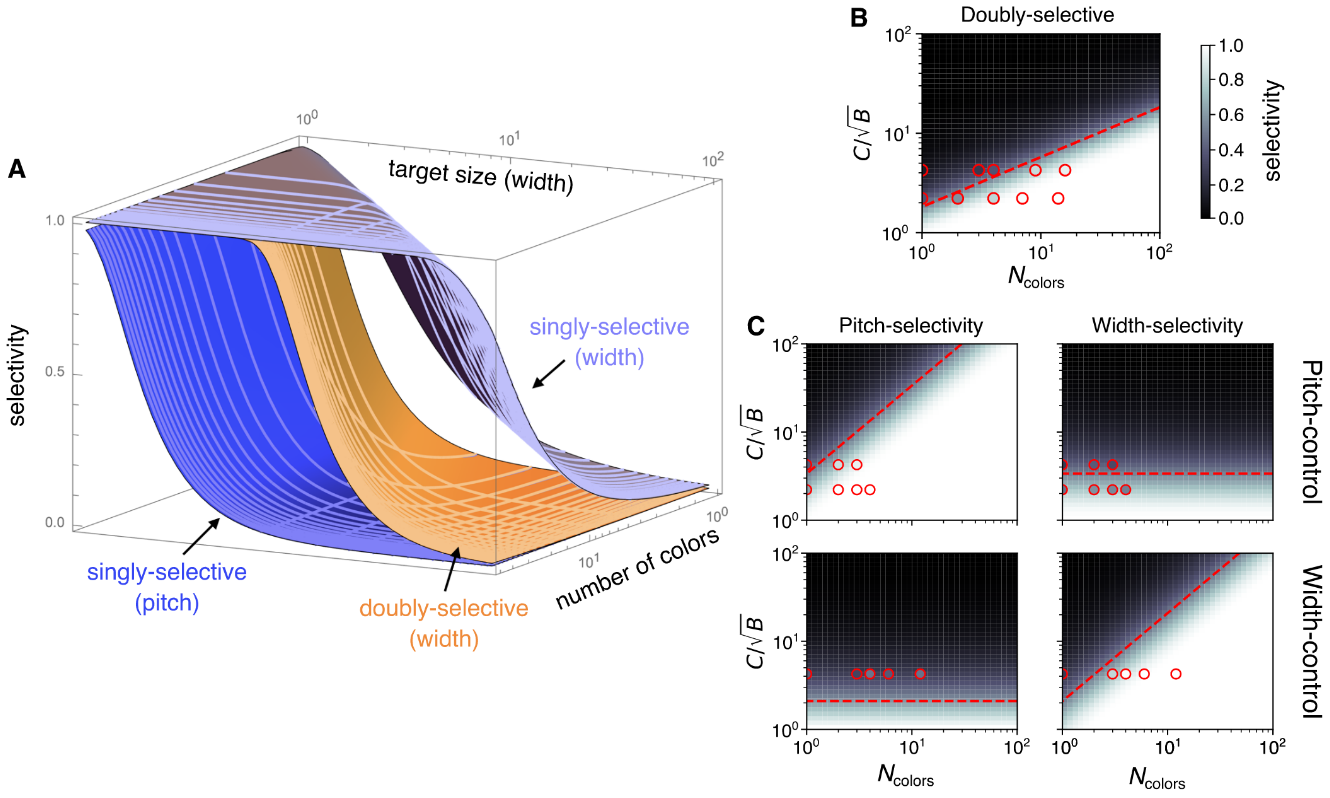

FIG. S10. **Selectivity in tubules for different strategies.** (**A**) Surface plot of selectivity of the tubule width plotted against the target tubule size and the number of colors. The orange surface corresponds to the scheme of targeting a single tubule state with an isotropic coloring pattern (called doubly-selective), as shown in the main text Fig. 2 and 3. For the width-control scheme (singly-selective), as shown in the main text Fig. 4, the light blue surface shows the selectivity of the tubule width while the dark blue shows the corresponding pitch selectivity. (**B**) Shows a color map of the doubly-selective strategy. Circle points show the experimental data shown from Fig. 3 and share the same colorbar as the theoretical points. (**C**) shows the pitch- (left) and width-selectivity (right) for both the pitch-control (top) and width-control (bottom) schemes. This demonstrates the trade-off that though the scaling of selectivity with the self-limited length is better while constraining just a single dimension of the assembly, one loses any improvement of selectivity in the other dimension of the assembly.

complexity can still be used to remove these types of structures if they are accessible.

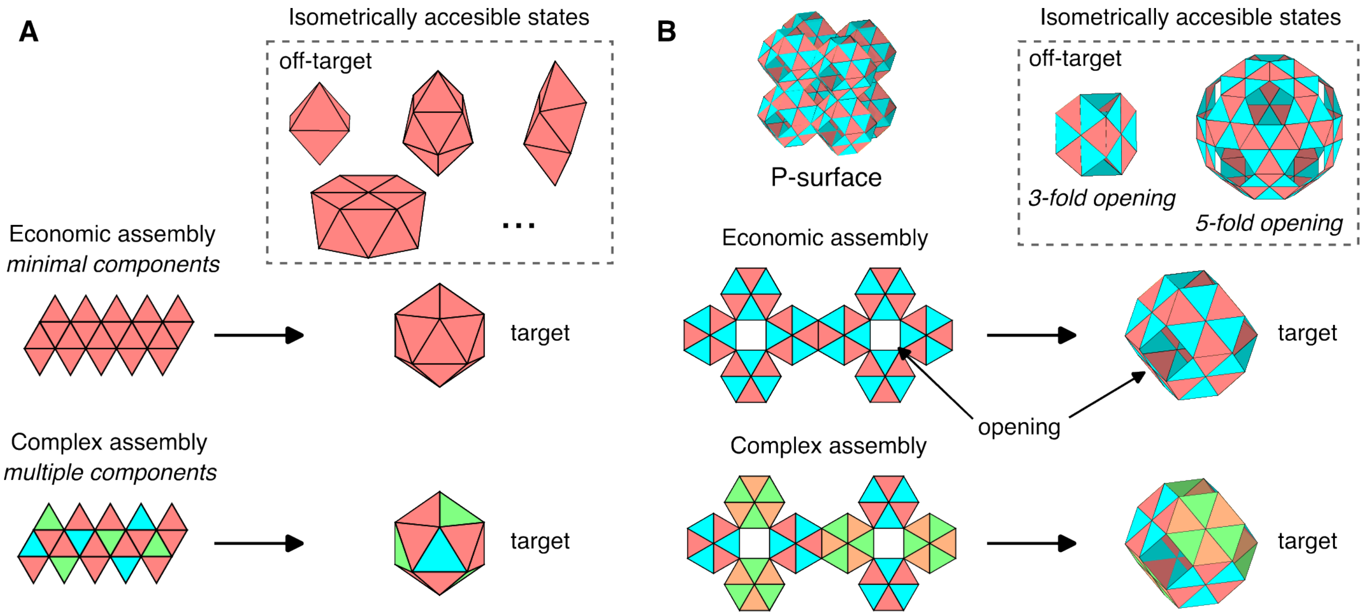

FIG. S11. **Extension of complex assembly to other geometries.** (**A**) Economical and complex tilings for icosahedral capsid assembly are shown. Due to the lack of interaction specificity of the economical assembly, the subunits that make it up could assemble into other closed structures. By increasing complexity, and thereby removing symmetries of the target structure, the target icosahedral capsid can be formed, but the off-target states cannot be accessed. (**B**) Economical and complex tilings for the assembly of a triangulated Schwarz P-surface (a triply periodic structure with cubic crystal symmetry). The unfolded tilings we show are for the primitive cell of this structure, which we call an 'octahedral cage'. These open octahedral cages can bind together to form a cubic lattice, as shown a the top left. Due to the symmetry of the interactions between the hexagonal facets, tetrahedral or icosahedral cages could assemble as well, inhibiting the formation of the higher-order P-surface. Note that the opening of each cage has different numbers of subunits around them. By increasing the complexity, right, we can remove odd-fold openings, as seen for the off-target structures.

TABLE S1: **Interaction sequence library.** A list of 72 unique sequences generated using the algorithm described in Suppl. Sec. S3, along with the complementary sequence and an estimate of their binding free energy.

Sequence	Compliment	ΔG [kcal/mol]	Sequence	Compliment	ΔG [kcal/mol]
ACTAGC	GCTAGT	-6.09	AGTTCC	GGAACT	-6.13
AGTTAC	GTAACT	-5.01	CGATTA	TAATCG	-4.42
TAGTCT	AGACTA	-4.53	ATTCTG	CAGAAT	-5.01
CGATGG	CCATCG	-6.61	ATTCAG	CTGAAT	-5.01
CCATTC	GAATGG	-5.61	CTTGAG	CTCAAG	-5.28
CTTGGT	ACCAAG	-5.92	GTAGAT	ATCTAC	-4.42
ATGCAC	GTGCAT	-6.73	GGATAA	TTATCC	-4.12
TCGACA	TGTCGA	-5.67	TCATCC	GGATGA	-5.43
TTGGAT	ATCCAA	-4.9	GGTATT	AATACC	-4.68
TCAGAC	GTCTGA	-5.43	GGTAAT	ATTACC	-4.68
GTCTAG	CTAGAC	-4.88	ACTGAG	CTCAGT	-5.85
TACCTT	AAGGTA	-4.79	AGAGAT	ATCTCT	-5.08
AGTCAG	CTGACT	-5.85	AGATAG	CTATCT	-4.42
CTCGAA	TTCGAG	-5.54	TTCCTG	CAGGAA	-5.36
TCCTTC	GAAGGA	-5.38	TTCCAT	ATGGAA	-4.9
GATCTT	AAGATC	-4.7	GATATG	CATATC	-4.09
CTGATC	GATCAG	-5.35	TTAACC	GGTTAA	-4.52
TCCACA	TGTGGA	-5.49	AACATT	AATGTT	-4.81
CAATAG	CTATTG	-4.16	TTGGCA	TGCCAA	-5.99
TGATTG	CAATCA	-4.57	GACCTC	GAGGTC	-6.33
CTAGGA	TCCTAG	-4.77	CCTATG	CATAGG	-5
CACATC	GATGTG	-5.66	CTTAGG	CCTAAG	-4.95
ACGAAG	CTTCGT	-6.29	TAACAG	CTGTTA	-4.24
ACCTGA	TCAGGT	-5.93	TCTTCT	AGAAGA	-4.59
ATGACA	TGTCAT	-5.14	GTACAT	ATGTAC	-4.73
TACAGG	CCTGTA	-5.08	ATAAGT	ACTTAT	-4.22
AACCTA	TAGGTT	-4.76	TTCAAT	ATTGAA	-4.06
GAGACA	TGTCTC	-5.29	CTTACT	AGTAAG	-4.49
GACAGA	TCTGTC	-5.29	AGTATC	GATACT	-4.75
CGTCCA	TGGACG	-6.69	GTATGT	ACATAC	-4.73
GCATCT	AGATGC	-6.09	ACAATT	AATTGT	-4.81
TATTCC	GGAATA	-4.26	ACTAAC	GTTAGT	-5.01
AGATTC	GAATCT	-5.03	CTTGTA	TACAAG	-4.24
TTCTCA	TGAGAA	-4.34	CTACAC	GTGTAG	-5.33
CTGTGA	TCACAG	-5.41	AAGTAG	CTACTT	-4.68
TCGTAC	GTACGA	-5.59	AACTAT	ATAGTT	-4.22

TABLE S2: **Side interactions for multicomponent assemblies.** A list of the set of six interaction sequences that make up a side interaction of a monomer and an estimate of their summed binding free energy. The first six sets of sequences are self-complimentary, e.g. Position 1 binds to Position 6, Position 2 binds to Position 5, and Position 3 binds to Position 4. For the rest of the sets, X is complimentary to X*.

Name	Position 1	Position 2	Position 3	Position 4	Position 5	Position 6	ΔG [kcal/mol]
sA side 1	ACTAGC	AGTTAC	TAGTCT	AGACTA	GTAACT	GCTAGT	-30.57
sA side 2	TTCAAT	CCATTC	CTTGGT	ACCAAG	GAATGG	ATTGAA	-31.42
sA side 3	TTAACC	TCGACA	TTGGAT	ATCCAA	TGTCGA	GGTTAA	-30.23
sB side 1	TCAGAC	GTCTAG	TACCTT	AAGGTA	CTAGAC	GTCTGA	-30.17
sB side 2	ATAAGT	CTCGAA	TCCTTC	GAAGGA	TTCGAG	ACTTAT	-30.14
sB side 3	GATCTT	CTGATC	TCCACA	TGTGGA	GATCAG	AAGATC	-31.27
A side 1	CAATAG	TGATTG	CTAGGA	CACATC	ACGAAG	ACCTGA	-31.38
A side 2	ATGACA	TACAGG	AACCTA	GAGACA	GACAGA	ACTAAC	-30.57
A side 3	GTACAT	AGTCAG	CGATGG	CTTACT	AGTATC	GTATGT	-31.16
A* side 1	TCAGGT	CTTCGT	GATGTG	TCCTAG	CAATCA	CTATTG	-31.38
A* side 2	GTTAGT	TCTGTC	TGTCTC	TAGGTT	CCTGTA	TGTCAT	-30.57
A* side 3	ACATAC	GATACT	AGTAAG	CCATCG	CTGACT	ATGTAC	-31.16
B side 1	GCATCT	TATTCC	AGATTC	TTCTCA	CTGTGA	TCGTAC	-30.72
B side 2	AGATAG	TTCCTG	TTCCAT	GATATG	ATGCAC	AACATT	-30.31
B side 3	ACAATT	CGTCCA	CTTGTA	CTACAC	GACAGA	AACTAT	-30.58
B* side 1	GTACGA	TCACAG	TGAGAA	GAATCT	GGAATA	AGATGC	-30.72
B* side 2	AATGTT	GTGCAT	CATATC	ATGGAA	CAGGAA	CTATCT	-30.31
B* side 3	ATAGTT	TCTGTC	GTGTAG	TACAAG	TGGACG	AATTGT	-30.58
C side 1	AGTTCC	CGATTA	ATTCTG	ATTCAG	CTTGAG	GTAGAT	-30.27
C side 2	GGATAA	TCATCC	GGTATT	GGTAAT	ACTGAG	AGAGAT	-29.84
C side 3	TTGGCA	GACCTC	CCTATG	CTTAGG	TAACAG	TCTTCT	-31.1
C* side 1	ATCTAC	CTCAAG	CTGAAT	CAGAAT	TAATCG	GGAACT	-30.27
C* side 2	ATCTCT	CTCAGT	ATTACC	AATACC	GGATGA	TTATCC	-29.84
C* side 3	AGAAGA	CTGTTA	CCTAAG	CATAGG	GAGGTC	TGCCAA	-31.1
D side 1	CACATC	CAATAG	ACGAAG	TGATTG	ACCTGA	CTAGGA	-31.38
D side 2	GAGACA	ATGACA	GACAGA	TACAGG	ACTAAC	AACCTA	-30.57
D side 3	CTTACT	GTACAT	AGTATC	AGTCAG	GTATGT	CGATGG	-31.16
D* side 1	TCCTAG	TCAGGT	CAATCA	CTTCGT	CTATTG	GATGTG	-31.38
D* side 2	TAGGTT	GTTAGT	CCTGTA	TCTGTC	TGTCAT	TGTCTC	-30.57
D* side 3	CCATCG	ACATAC	CTGACT	GATACT	ATGTAC	AGTAAG	-31.16
E side 1	TTCTCA	GCATCT	CTGTGA	TATTCC	TCGTAC	AGATTC	-30.72
E side 2	GATATG	AGATAG	ATGCAC	TTCCTG	AACATT	TTCCAT	-30.31
E side 3	CTACAC	ACAATT	GACAGA	CGTCCA	AACTAT	CTTGTA	-30.58
E* side 1	GAATCT	GTACGA	GGAATA	TCACAG	AGATGC	TGAGAA	-30.72
E* side 2	ATGGAA	AATGTT	CAGGAA	GTGCAT	CTATCT	CATATC	-30.31
E* side 3	TACAAG	ATAGTT	TGGACG	TCTGTC	AATTGT	GTGTAG	-30.58
F side 1	ATTCAG	AGTTCC	CTTGAG	CGATTA	GTAGAT	ATTCTG	-30.27
F side 2	GGTAAT	GGATAA	ACTGAG	TCATCC	AGAGAT	GGTATT	-29.84
F side 3	CTTAGG	TTGGCA	TAACAG	GACCTC	TCTTCT	CCTATG	-31.1
F* side 1	CAGAAT	ATCTAC	TAATCG	CTCAAG	GGAACT	CTGAAT	-30.27
F* side 2	AATACC	ATCTCT	GGATGA	CTCAGT	TTATCC	ATTACC	-29.84
F* side 3	CATAGG	AGAAGA	GAGGTC	CTGTTA	TGCCAA	CCTAAG	-31.1
G side 1	ACGAAG	CTAGGA	ACCTGA	CAATAG	CACATC	TGATTG	-31.38
G side 2	GACAGA	AACCTA	ACTAAC	ATGACA	GAGACA	TACAGG	-30.57
G side 3	AGTATC	CGATGG	GTATGT	GTACAT	CTTACT	AGTCAG	-31.16
G* side 1	CAATCA	GATGTG	CTATTG	TCAGGT	TCCTAG	CTTCGT	-31.38
G* side 2	CCTGTA	TGTCTC	TGTCAT	GTTAGT	TAGGTT	TCTGTC	-30.57
G* side 3	CTGACT	AGTAAG	ATGTAC	ACATAC	CCATCG	GATACT	-31.16
H side 1	CTGTGA	AGATTC	TCGTAC	GCATCT	TTCTCA	TATTCC	-30.72
H side 2	ATGCAC	TTCCAT	AACATT	AGATAG	GATATG	TTCCTG	-30.31
H side 3	GACAGA	CTTGTA	AACTAT	ACAATT	CTACAC	CGTCCA	-30.58
H* side 1	GGAATA	TGAGAA	AGATGC	GTACGA	GAATCT	TCACAG	-30.72
H* side 2	CAGGAA	CATATC	CTATCT	AATGTT	ATGGAA	GTGCAT	-30.31
H* side 3	TGGACG	GTGTAG	AATTGT	ATAGTT	TACAAG	TCTGTC	-30.58
I side 1	CTTGAG	ATTCTG	GTAGAT	AGTTCC	ATTCAG	CGATTA	-29.84
I side 2	ACTGAG	GGTATT	AGAGAT	GGATAA	GGTAAT	TCATCC	-31.1
I side 3	TAACAG	CCTATG	TCTTCT	TTGGCA	CTTAGG	GACCTC	-31.42

Name	Position 1	Position 2	Position 3	Position 4	Position 5	Position 6	ΔG [kcal/mol]
I* side 1	TAATCG	CTGAAT	GGAACT	ATCTAC	CAGAAT	CTCAAG	-29.84
I* side 2	GGATGA	ATTACC	TTATCC	ATCTCT	AATACC	CTCAGT	-31.1
I* side 3	GAGGTC	CCTAAG	TGCCAA	AGAAGA	CATAGG	CTGTTA	-31.42

TABLE S3: **Subunit interactions for isotropic colorings.** This table enumerates the different side interactions from Table S2 used to generate the colorings used in Fig. 2 and 3 in the main text for the (6,0) tubule.

Number of subunits	Subunit ID	Side 1 strands	Side 2 strands	Side 3 strands	Number of subunits	Subunit ID	Side 1 strands	Side 2 strands	Side 3 strands
N=1	1	sA	sA	sA	N=14	1	A	A	A
N=2	1	sA	A	A		2	B	B	A*
	2	A	A*	sA		3	C	A*	B
N=4	1	sA	A	A		4	B*	C	C
	2	sB	B	A*		5	C*	D	D
	3	A	A*	sA		6	A*	B*	E
	4	A*	B*	sB		7	D	E	C*
N=7	1	A	A	sA		8	D*	C*	D*
	2	A*	B	A		9	E	D*	sA
	3	B	A*	A*		10	F	F	E*
	4	C	B*	B		11	E*	G	F
	5	B*	C	B*		12	F*	E*	F*
	6	C*	sA	C		13	G	F*	sB
	7	sA	C*	C*		14	G*	G*	B*

TABLE S4: **Subunit interactions for isotropic colorings.** This table enumerates the different side interactions from Table S2 used to generate the colorings used in Fig. 2 and 3 in the main text for the (10,0) tubule.

Number of subunits	Subunit ID	Side 1 strands	Side 2 strands	Side 3 strands	Number of subunits	Subunit ID	Side 1 strands	Side 2 strands	Side 3 strands
N=1	1	sA	sA	sA	N=16	1	A	A	A
N=3	1	sA	A	A		2	B	B	A*
	2	A	A*	sA		3	B*	C	B
	3	A*	sA	A*		4	C	D	C
N=4	1	A	A	A		5	A*	B*	D
	2	A*	B	B		6	D	E	B*
	3	B	A*	B*		7	E	C*	C*
	4	B*	B*	A*		8	F	F	D*
N=9	1	sA	A	A		9	D*	G	E
	2	B	A*	B		10	E*	D*	F
	3	C	B	A*		11	F*	E*	G
	4	B*	sA	C		12	G	H	E*
	5	A	D	B*		13	G*	G*	F*
	6	C*	C	sA		14	H	F*	G*
	7	A*	B*	D		15	C*	A*	H
	8	D	C*	C*		16	H*	H*	H*
	9	D*	D*	D*					

TABLE S5: **Subunit interactions for linear tubule colorings.** This table enumerates the different side interactions from Table S2 used to generate the colorings used in Fig. 4 and 5 in the main text. For the pitch- and width-controlled tilings, we use the "Linear tiling monomers" shown in the table below. These monomers have a repetitive nature to their interactions and the linear coloring can be terminated at any number of colors in the tiling. If the number of colors is even, we replace the final monomer with the "Even periodic" monomer, where X* matches the interaction of the replaced monomer, i.e. if we made an 8-color linear tiling then we would use the "Linear tiling monomers" 1 to 7 and an "Even periodic" monomer with X* being D*, following the subunit ID 8 pattern. If the number of colors is odd, we replace the final monomer with the "Odd periodic" interactions, swapping X* to the interaction of the replaced monomer. Different permutations of the side interactions can orient the linear colorings along different lattice directions. Pitch-controlled tubules have side identifications as shown in the table, while width-controlled tubules swap the Side 2 and Side 3 interactions. Similarly, to make length-controlled tubules we can take a linear tiling and make the final monomer a "Capping monomer", that has no interaction on its side 3.

Linear tiling monomers				Capping Monomers			
Subunit ID	Side 1 strands	Side 2 strands	Side 3 strands	Subunit ID	Side 1 strands	Side 2 strands	Side 3 strands
1	sA	sA	A	1-cap	sA	sA	Passive
2	A	A	A*				
3	A*	A*	B	3-cap	A*	A*	Passive
4	B	B	B*				
5	B*	B*	C				
6	C	C	C*				
7	C*	C*	D	7-cap	C*	C*	Passive
8	D	D	D*				
9	D*	D*	E				
10	E	E	E*				
11	E*	E*	F				
12	F	F	F*				
13	F*	F*	G				
14	G	G	G*				
15	G*	G*	H				
16	H	H	H*				
17	H*	H*	I				
18	I	I	I*	19-cap	I*	I*	Passive
Even periodic	sB	sB	X*				
Odd periodic	X*	X*	sA				

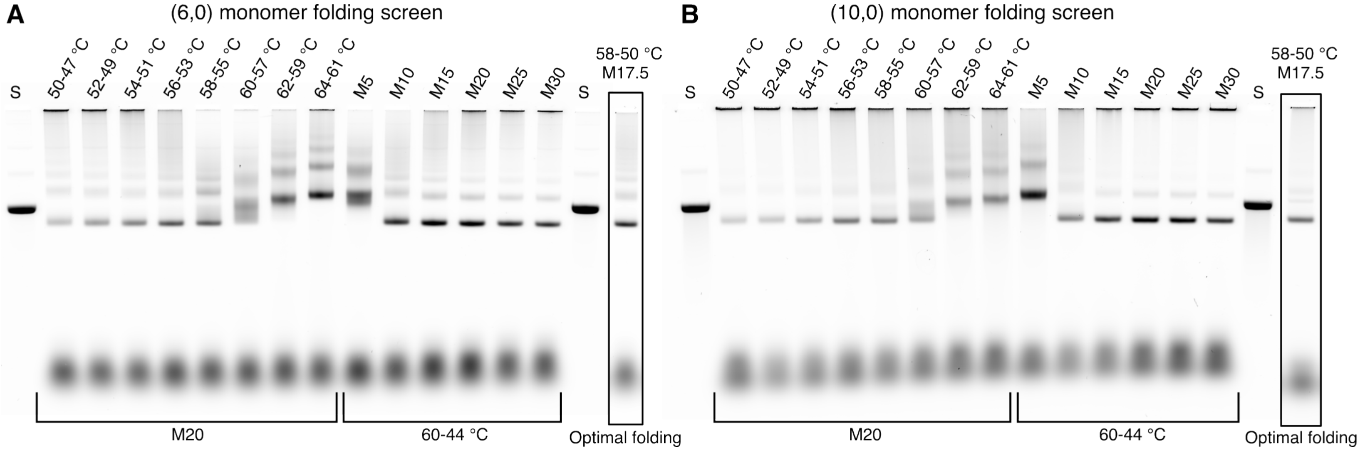

FIG. S12. **Folding screens for DNA origami monomers.** Here are images of gels for the initial folding screens as well as a lane, taken from a different gel, of the optimal folding condition we used for both the (**A**) (6,0) monomer and the (**B**) (10,0) monomer. Labels above gel lanes denote folding temperatures with steps of 1 °C/hr or $MgCl_2$ concentration where MX means X mM $MgCl_2$. Scaffold DNA has been run in lanes labeled by S.

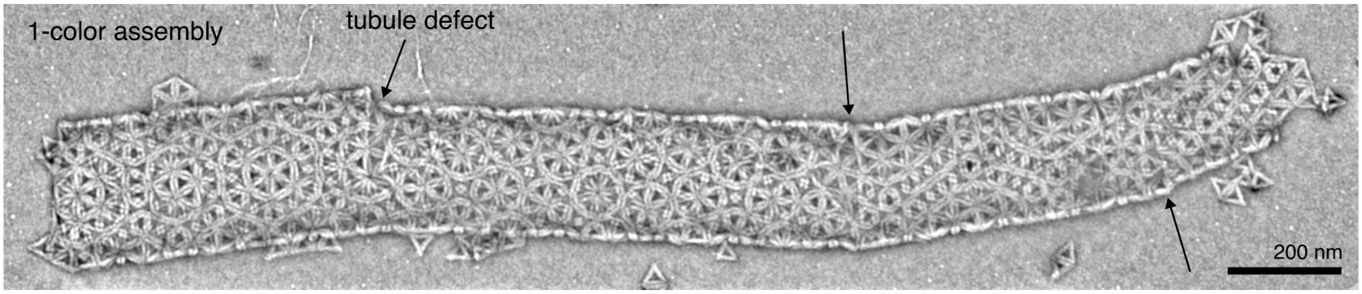

FIG. S13. **Common defect mode in low complexity tubule assembly.** Here is an example of a single-component tubule grown near the melting transition. The arrows point towards locations along the tubule where there is a defect between two different closure types. The slower growth occurs, i.e. at higher temperatures, the propensity to form defects decreases. This image has been bandpass filtered

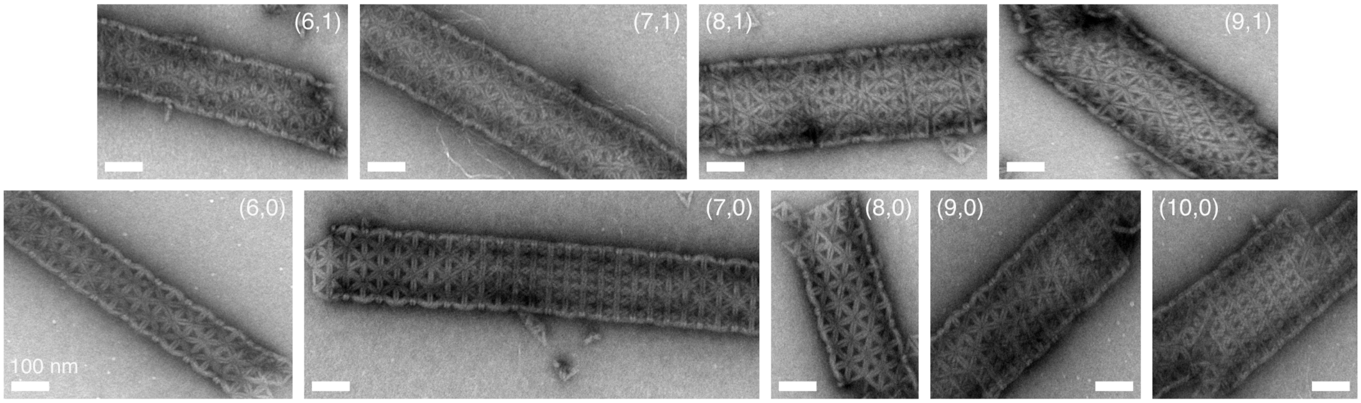

FIG. S14. **Examples of different assembly states with the (6,0) monomer.** All images are labeled with their (m, n) type and the scale bars are 100 nm.

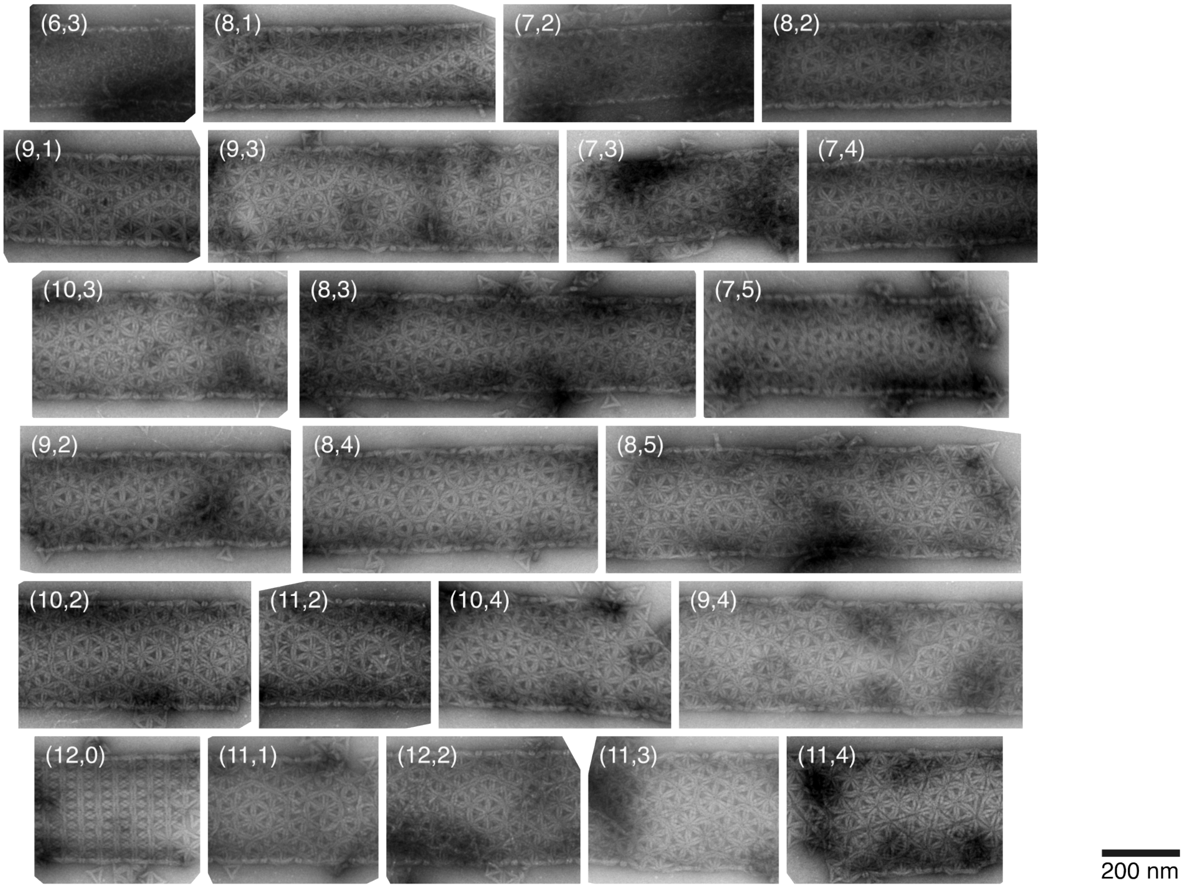

FIG. S15. **Examples of different assembly states with the (10,0) monomer.** All images are labeled with their (m, n) type and share the same scale bar, 200 nm.

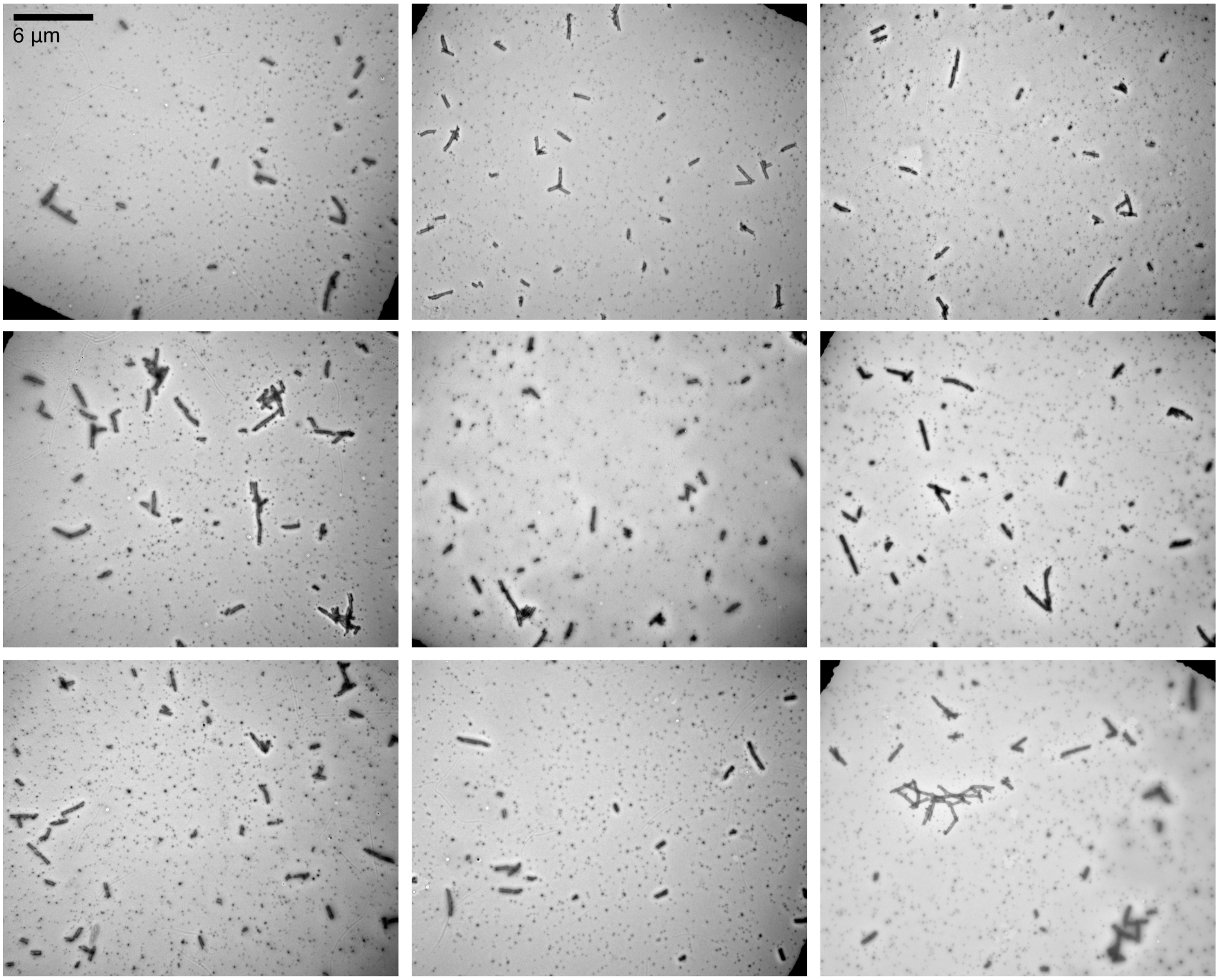

FIG. S16. **Overview of 1-color assemblies with the (6,0) monomer.** The TEM images primarily show tubules and unbound monomers/small oligomers. While there are a few small clusters of tubules, we hypothesize that those clusters form during grid preparation. Noteably we do not see any large, random aggregates. All images share the same scale bar.

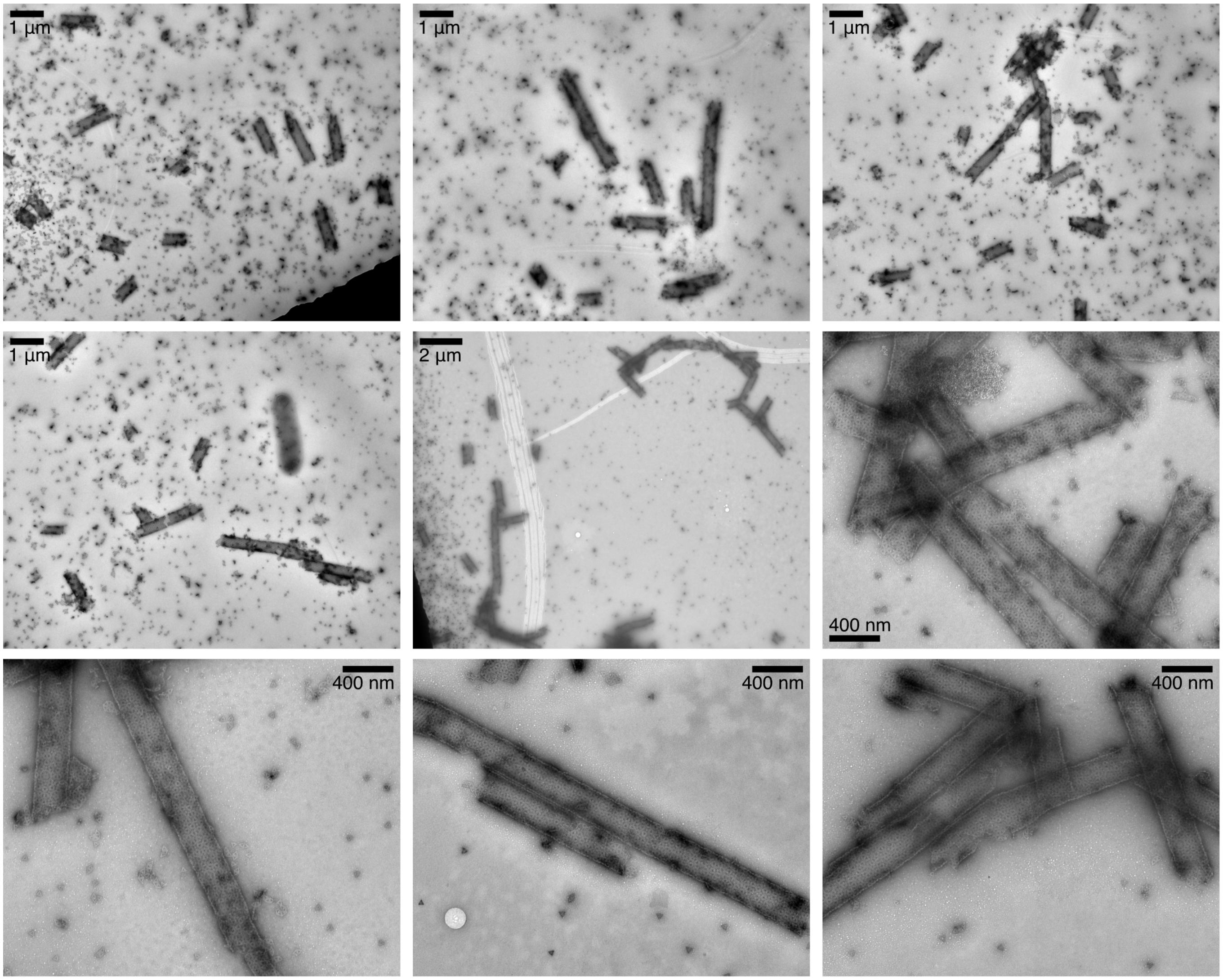

FIG. S17. **Overview of 9-color assemblies with the (10,0) monomer.** The TEM images primarily show tubules and unbound monomers/small oligomers. While there are a few small clusters of tubules, we hypothesize that those clusters form during grid preparation. Noteably we do not see any large, random aggregates. There is a large fraction of monomers and small clusters in this 9-color assembly compared to the 1-color assembly shown in Fig. S16.

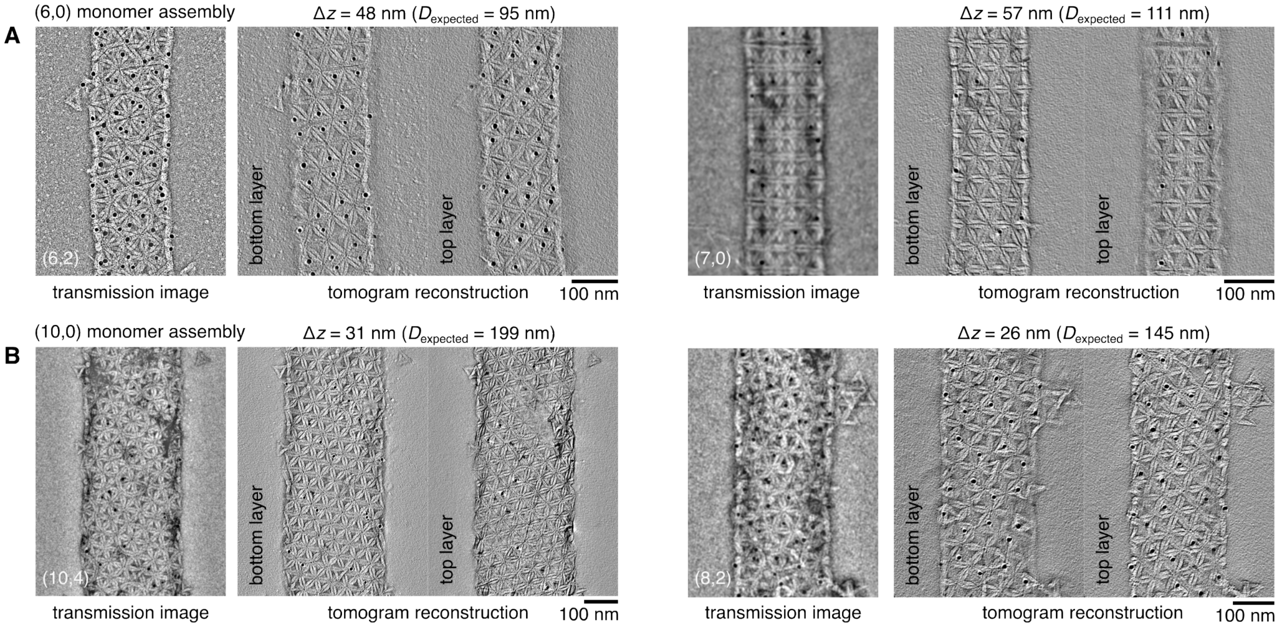

FIG. S18. **Tomogram reconstructions of tubules.** Here we show the top and bottom layers of tomographic reconstructions for tubules from (**A**) the (6,0) monomer and (**B**) the (10,0) monomer. From these reconstructions we find that the difference in height between the top and bottom layers is on the order of the size of a monomer, implying the tubules are flattened on the TEM grid. Transmission images have been bandpass filtered. The height of the tomograms, Δz, as well as the expected diameter for the unflattened tubule, D_{expected}, are given above each image.

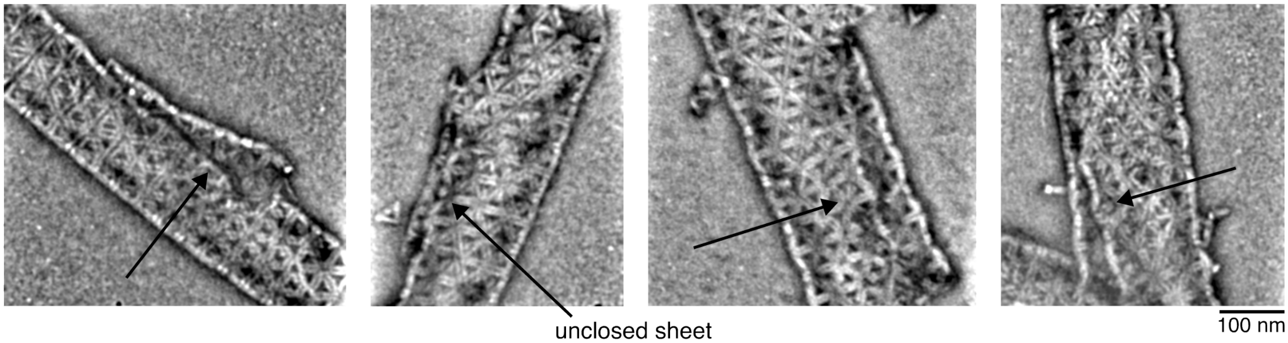

FIG. S19. **Scroll defects in overly complex assembly.** In the 14-color assembly with the (6,0) monomer we occasionally see scroll defects, where the sheet grows wider than the preferred tubule circumference. Arrows point towards regions where we can see two "walls" of the tubule, where the TEM looks brighter. Images have been bandpass filtered.

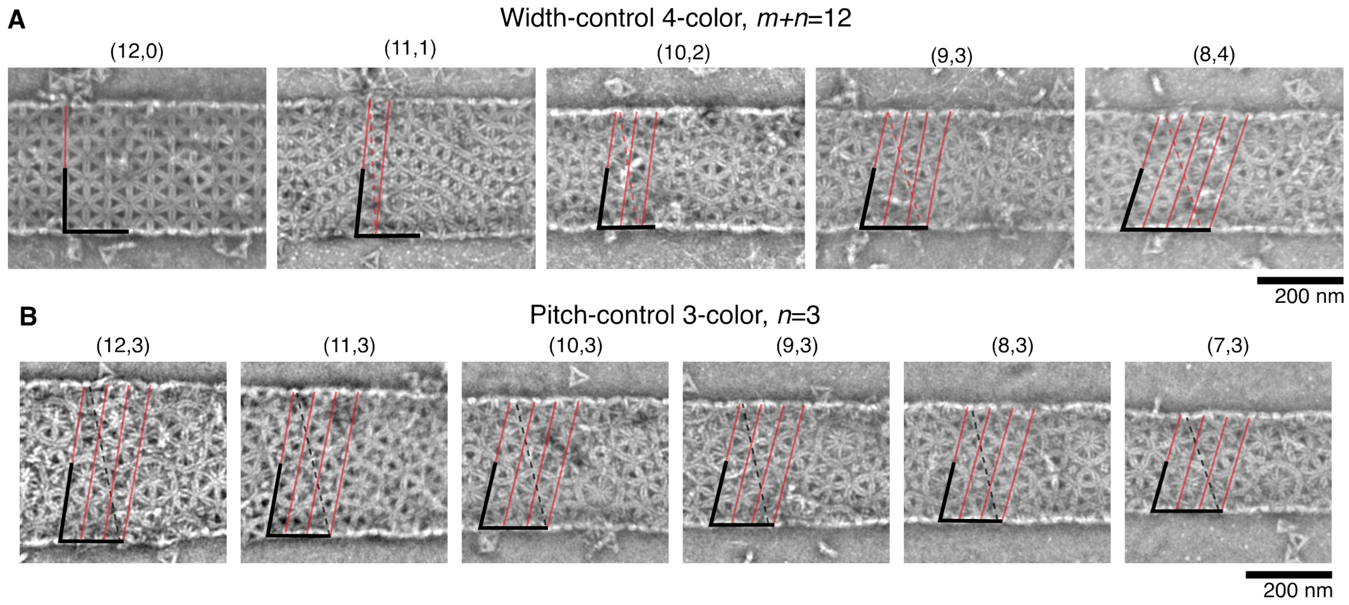

FIG. S20. **Gallery of width and pitch controlled tubules** (**A**) Tubules with $m + n = 12$ from a 4-color width-controlled experiment. Tubules have nearly constant width, but varying pitch. (**B**) Tubules with $n = 3$ from a 3-color pitch-controlled experiment. Tubules have constant pitch, but varying width. Both sets of experiments come from the distributions shown in Fig. 4A in the main text. Images have been bandpass filtered.

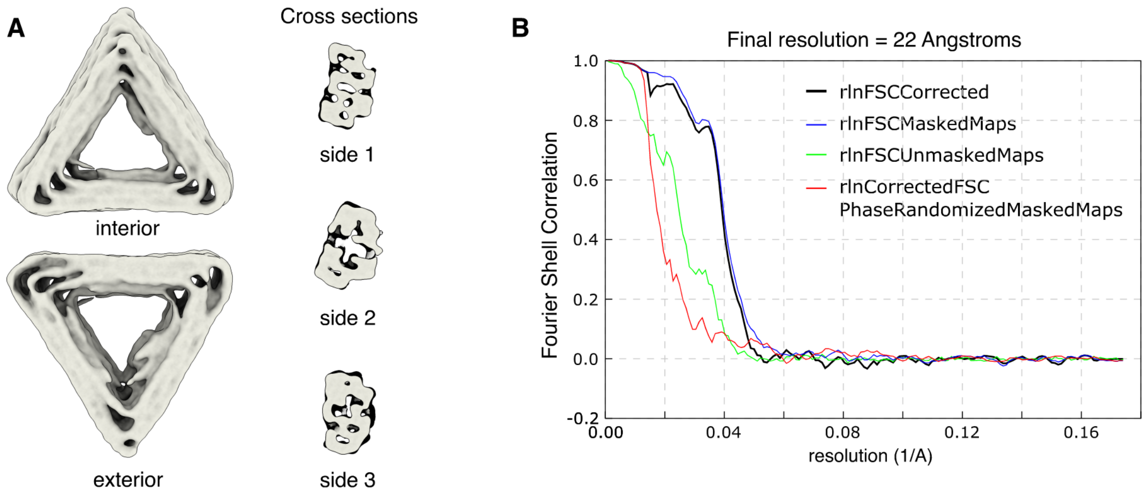

FIG. S21. **Cryo-EM reconstruction of (6,0) monomer.** (**A**) Views of the DNA origami monomer from the interior or exterior with respect to the tubules that form. Cross-sectional slices of the middle of each side. (**B**) Plot of the FSC curves used to estimate the resolution of the monomer reconstruction.

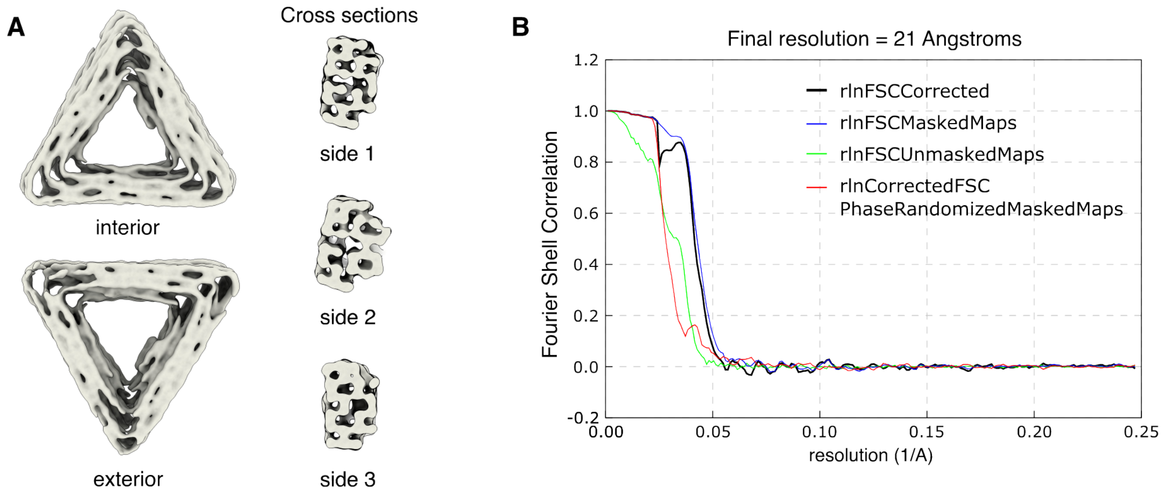

FIG. S22. **Cryo-EM reconstruction of (10,0) monomer.** (**A**) Views of the DNA origami monomer from the interior or exterior with respect to the tubules that form. Cross-sectional slices of the middle of each side. (**B**) Plot of the FSC curves used to estimate the resolution of the monomer reconstruction.

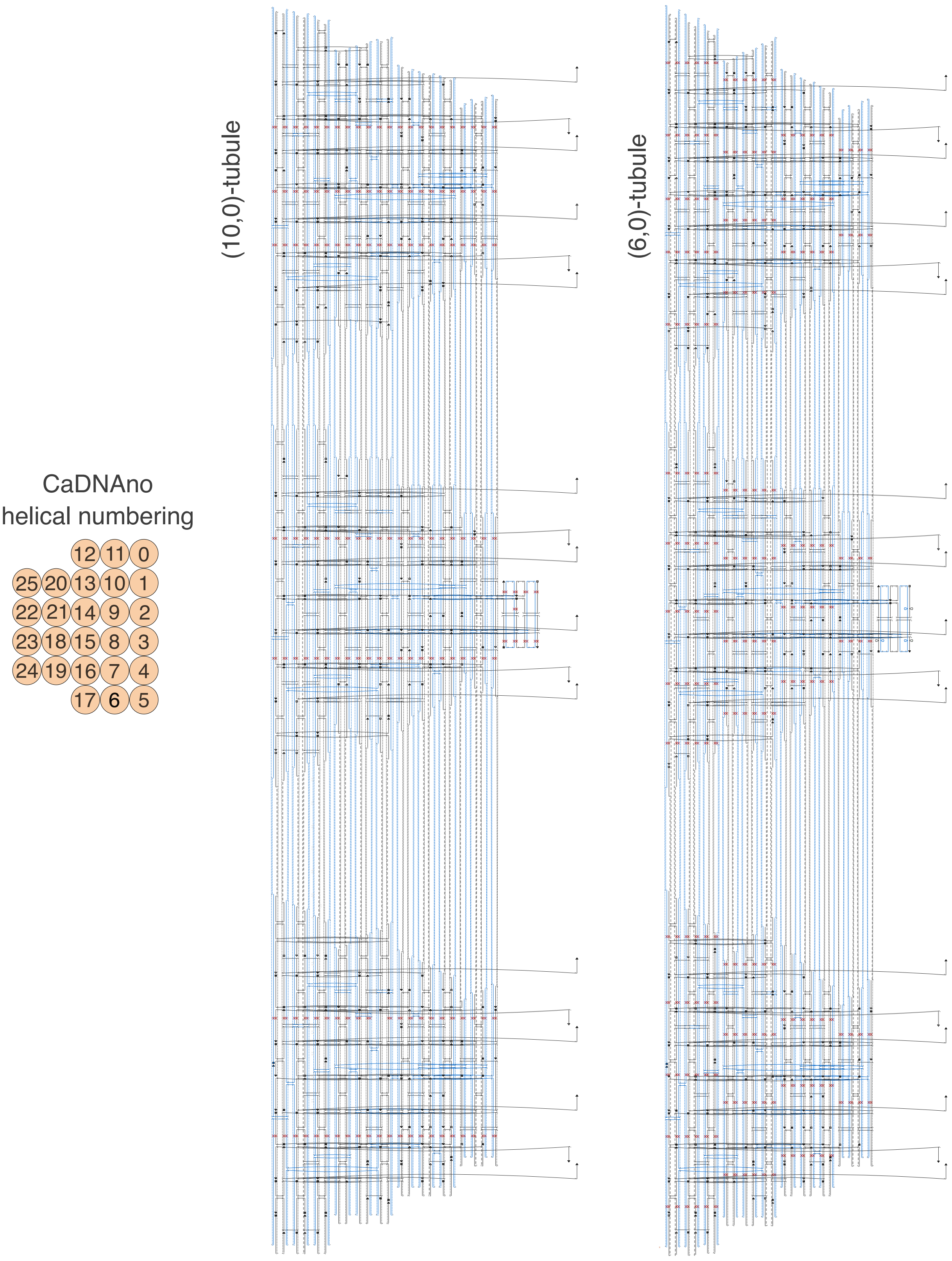

FIG. S23. **caDNAno design of DNA origami monomer with helical numbering.**

REFERENCES AND NOTES

1. G. M. Whitesides, B. Grzybowski, Self-assembly at all scales. *Science* **295**, 2418–2421 (2002).

2. D. J. Kraft, J. Groenewold, W. K. Kegel, Colloidal molecules with well-controlled bond angles. *Soft Matter* **5**, 3823–3826 (2009).

3. S. Sacanna, M. Korpics, K. Rodriguez, L. Colón-Meléndez, S.-H. Kim, D. J. Pine, G.-R. Yi, Shaping colloids for self-assembly. *Nat. Commun.* **4**, 1688 (2013).

4. Y. Wang, Y. Wang, X. Zheng, G.-R. Yi, S. Sacanna, D. J. Pine, M. Weck, Three-dimensional lock and key colloids. *J. Am. Chem. Soc.* **136**, 6866–6869 (2014).

5. Q. Chen, S. C. Bae, S. Granick, Directed self-assembly of a colloidal kagome lattice. *Nature* **469**, 381–384 (2011).

6. M. He, J. P. Gales, É. Ducrot, Z. Gong, G.-R. Yi, S. Sacanna, D. J. Pine, Colloidal diamond. *Nature* **585**, 524–529 (2020).

7. A. Hensley, W. M. Jacobs, W. B. Rogers, Self-assembly of photonic crystals by controlling the nucleation and growth of DNA-coated colloids. *Proc. Natl. Acad. Sci. U.S.A.* **119**, e2114050118 (2022).

8. A. Hensley, T. E. Videbæk, H. Seyforth, W. M. Jacobs, W. B. Rogers, Macroscopic photonic single crystals via seeded growth of DNA-coated colloids. *Nat. Commun.* **14**, 4237 (2023).

9. D. Zerrouki, J. Baudry, D. Pine, P. Chaikin, J. Bibette, Chiral colloidal clusters. *Nature* **455**, 380–382 (2008).

10. S. Sacanna, W. T. Irvine, P. M. Chaikin, D. J. Pine, Lock and key colloids. *Nature* **464**, 575–578 (2010).

11. Q. Chen, J. K. Whitmer, S. Jiang, S. C. Bae, E. Luijten, S. Granick, Supracolloidal reaction kinetics of Janus spheres. *Science* **331**, 199–202 (2011).

12. J. R. Wolters, G. Avvisati, F. Hagemans, T. Vissers, D. J. Kraft, M. Dijkstra, W. K. Kegel, Self-assembly of "Mickey Mouse" shaped colloids into tube-like structures: Experiments and simulations. *Soft Matter* **11**, 1067–1077 (2015).

13. G. Tikhomirov, P. Petersen, L. Qian, Triangular DNA origami tilings. *J. Am. Chem. Soc.* **140**, 17361–17364 (2018).

14. J. S. Oh, S. Lee, S. C. Glotzer, G.-R. Yi, D. J. Pine, Colloidal fibers and rings by cooperative assembly. *Nat. Commun.* **10**, 3936 (2019).

15. J. S. Kahn, B. Minevich, A. Michelson, H. Emamy, K. Kisslinger, S. Xiang, S. K. Kumar, O. Gang, Encoding hierarchical 3D architecture through inverse design of programmable bonds. ChemRxiv 10.26434/chemrxiv-2022-xwbst (2022).

16. D. L. Caspar, A. Klug, Physical principles in the construction of regular viruses. *Cold Spring Harb. Symp. Quant. Biol.* **27**, 1–24 (1962).

17. K. Michielsen, D. G. Stavenga, Gyroid cuticular structures in butterfly wing scales: Biological photonic crystals. *J. R. Soc. Interface* **5**, 85–94 (2008).

18. K. F. Wagenbauer, C. Sigl, H. Dietz, Gigadalton-scale shape-programmable DNA assemblies. *Nature* **552**, 78–83 (2017).

19. C. Sigl, E. M. Willner, W. Engelen, J. A. Kretzmann, K. Sachenbacher, A. Liedl, F. Kolbe, F. Wilsch, S. A. Aghvami, U. Protzer, M. F. Hagan, S. Fraden, H. Dietz, Programmable icosahedral shell system for virus trapping. *Nat. Mater.* **20**, 1281–1289 (2021).

20. D. Hayakawa, T. E. Videbæk, D. M. Hall, H. Fang, C. Sigl, E. Feigl, H. Dietz, S. Fraden, M. F. Hagan, G. M. Grason, W. B. Rogers, Geometrically programmed self-limited assembly of tubules using DNA origami colloids. *Proc. Natl. Acad. Sci. U.S.A.* **119**, e2207902119 (2022).

21. M. F. Hagan, G. M. Grason, Equilibrium mechanisms of self-limiting assembly. *Rev. Mod. Phys.* **93**, 025008 (2021).

22. W. Helfrich, Size distributions of vesicles: The role of the effective rigidity of membranes. *J. Phys. (Paris)* **47**, 321–329 (1986).

23. K. J. Böhm, W. Vater, H. Fenske, E. Unger, Effect of microtubule-associated proteins on the protofilament number of microtubules assembled in vitro. *Biochim. Biophys. Acta* **800**, 119–126 (1984).

24. D. Chrétien, R. H. Wade, New data on the microtubule surface lattice. *Biol. Cell* **71**, 161–174 (1991).

25. J. Roostalu, T. Surrey, Microtubule nucleation: Beyond the template. *Nat. Rev. Mol. Cell Biol.* **18**, 702–710 (2017).

26. C. Uetrecht, N. R. Watts, S. J. Stahl, P. T. Wingfield, A. C. Steven, A. J. R. Heck, Subunit exchange rates in Hepatitis B virus capsids are geometry- and temperature-dependent. *Phys. Chem. Chem. Phys.* **12**, 13368–13371 (2010).

27. P. W. K. Rothemund, A. Ekani-Nkodo, N. Papadakis, A. Kumar, D. K. Fygenson, E. Winfree, Design and characterization of programmable DNA nanotubes. *J. Am. Chem. Soc.* **126**, 16344–16352 (2004)

28. A. M. Mohammed, R. Schulman, Directing self-assembly of DNA nanotubes using programmable seeds. *Nano Lett.* **13**, 4006–4013 (2013).

29. Q. M. Dowling, Y.-J. Park, N. Gerstenmaier, E. C. Yang, A. Wargacki, Y. Hsia, C. N. Fries, R. Ravichandran, C. Walkey, A. Burrell, D. Veesler, D. Baker, N. P. King, Hierarchical design of pseudosymmetric protein nanoparticles. bioRxiv 545393 [Preprint] (2023). https://doi.org/10.1101/2023.06.16.545393.

30. C. Karfusehr, M. Eder, F. C. Simmel, Self-assembled cell-scale containers made from DNA origami membranes. bioRxiv 579479 [Preprint] (2024). https://doi.org/10.1101/2024.02.09.579479.

31. F. M. Gartner, I. R. Graf, P. Wilke, P. M. Geiger, E. Frey, Stochastic yield catastrophes and robustness in self-assembly. *eLife* **9**, e51020 (2020).

32. T. E. Videbæk, H. Fang, D. Hayakawa, B. Tyukodi, M. F. Hagan, W. B. Rogers, Tiling a tubule: How increasing complexity improves the yield of self-limited assembly. *J. Phys. Condens. Matter* **34**, 134003 (2022).

33. H. Sui, K. H. Downing, Structural basis of interprotofilament interaction and lateral deformation of microtubules. *Structure* **18**, 1022–1031 (2010).

34. A. Murugan, J. Zou, M. P. Brenner, Undesired usage and the robust self-assembly of heterogeneous structures. *Nat. Commun.* **6**, 6203 (2015).

35. C. M. Wintersinger, D. Minev, A. Ershova, H. M. Sasaki, G. Gowri, J. F. Berengut, F. E. Corea-Dilbert, P. Yin, W. M. Shih, Multi-micron crisscross structures grown from DNA-origami slats. *Nat. Nanotechnol.* **18**, 281–289 (2023).

36. D. Hayakawa, T. E. Videbæk, G. M. Grason, W. B. Rogers, Symmetry-guided inverse design of self-assembling multiscale DNA origami tilings. arXiv:2403.19518 (2024).

37. N. C. Seeman, De novo design of sequences for nucleic acid structural engineering. *J. Biomol. Struct. Dyn.* **8**, 573–581 (1990).

38. K.-T. Wu, L. Feng, R. Sha, R. Dreyfus, A. Y. Grosberg, N. C. Seeman, P. M. Chaikin, Polygamous particles. *Proc. Natl. Acad. Sci. U.S.A.* **109**, 18731–18736 (2012).

39. G. Tikhomirov, P. Petersen, L. Qian, Fractal assembly of micrometre-scale DNA origami arrays with arbitrary patterns. *Nature* **552**, 67–71 (2017).

40. K. F. Wagenbauer, F. A. Engelhardt, E. Stahl, V. K. Hechtl, P. Stömmer, F. Seebacher, L. Meregalli, P. Ketterer, T. Gerling, H. Dietz, How we make DNA origami. *Chembiochem* **18**, 1873–1885 (2017).

41. W. Helfrich, J. Prost, Intrinsic bending force in anisotropic membranes made of chiral molecules. *Phys. Rev. A* **38**, 3065–3068 (1988).

42. H. Fang, B. Tyukodi, W. B. Rogers, M. F. Hagan, Polymorphic self-assembly of helical tubules is kinetically controlled. *Soft Matter* **18**, 6716–6728 (2022).

43. C. M. Duque, D. M. Hall, B. Tyukodi, M. F. Hagan, C. D. Santangelo, G. M. Grason, Limits of economy and fidelity for programmable assembly of size-controlled triply-periodic polyhedra. arXiv:2309.04632 (2023).

44. M. H. Huntley, A. Murugan, M. P. Brenner, Information capacity of specific interactions. *Proc. Natl. Acad. Sci. U.S.A.* **113**, 5841–5846 (2016).

45. H. Tanaka, T. Dotera, S. T. Hyde, Programmable self-assembly of nanoplates into bicontinuous nanostructures. *ACS Nano* **17**, 15371–15378 (2023).

46. Z. Zeravcic, V. N. Manoharan, M. P. Brenner, Size limits of self-assembled colloidal structures made using specific interactions. *Proc. Natl. Acad. Sci. U.S.A.* **111**, 15918–15923 (2014).

47. J. Russo, F. Romano, L. Kroc, F. Sciortino, L. Rovigatti, P. Šulc, SAT-assembly: A new approach for designing self-assembling systems. *J. Phys. Condens. Matter* **34**, 354002 (2022).

48. D. E. Pinto, P. Šulc, F. Sciortino, J. Russo, Design strategies for the self-assembly of polyhedral shells. *Proc. Natl. Acad. Sci. U.S.A.* **120**, e2219458120 (2023).
49. W. M. Jacobs, A. Reinhardt, D. Frenkel, Rational design of self-assembly pathways for complex multicomponent structures. *Proc. Natl. Acad. Sci. U.S.A.* **112**, 6313–6318 (2015).
50. A. I. Curatolo, O. Kimchi, C. P. Goodrich, M. P. Brenner, The assembly yield of complex, heterogeneous structures: A computational toolbox. bioRxiv 497606 [Preprint] (2022). https://doi.org/10.1101/2022.06.26.497606.
51. A. Bupathy, D. Frenkel, S. Sastry, Temperature protocols to guide selective self-assembly of competing structures. *Proc. Natl. Acad. Sci. U.S.A.* **119**, e2119315119 (2022).
52. T.-J. Fu, N. C. Seeman, DNA double-crossover molecules. *Biochemistry* **32**, 3211–3220 (1993).
53. P. Yin, R. F. Hariadi, S. Sahu, H. M. T. Choi, S. H. Park, T. H. LaBean, J. H. Reif, Programming DNA tube circumferences. *Science* **321**, 5890, 824–826 (2008).
54. Y. Zhang, X. Chen, G. Kang, R. Peng, V. Pan, R. Sundaresan, P. Wang, Y. Ke, Programming DNA tube circumference by tile offset connection. *J. Am. Chem. Soc.* **141**, 19529–19532 (2019).
55. J. Sharma, R. Chhabra, A. Cheng, J. Brownell, Y. Liu, H. Yan, Control of self-assembly of DNA tubules through integration of gold nanoparticles. *Science* **323**, 112–116 (2009).
56. Y. Liu, J. Cheng, S. Fan, H. Ge, T. Luo, L. Tang, B. Ji, C. Zhang, D. Cui, Y. Ke, J. Song, Modular reconfigurable DNA origami: From two-dimensional to three-dimensional structures, *Angew. Chem. Int. Ed.* **59**, 23277–23282 (2020).
57. Y. Zhang, V. Pan, X. Li, X. Yang, H. Li, P. Wang, Y. Ke, Dynamic DNA structures. *Small* **15**, e1900228 (2019).

58. J. Song, Z. Li, P. Wang, T. Meyer, C. Mao, Y. Ke, Reconfiguration of DNA molecular arrays driven by information relay. *Science* **357**, eaan3377 (2017).

59. M. Kim, C. Lee, K. Jeon, J. Y. Lee, Y.-J. Kim, J. G. Lee, H. Kim, M. Cho, D.-N. Kim, Harnessing a paper-folding mechanism for reconfigurable DNA origami. *Nature* **619**, 78–86 (2023).

60. E. Poppleton, A. Mallya, S. Dey, J. Joseph, P. Šulc, Nanobase.org: A repository for DNA and RNA nanostructures. *Nucleic Acids Res.* **50**, D246–D252 (2022).

61. S. Sun, S. Yang, H. L. Xin, D. Nykypanchuk, M. Liu, H. Zhang, O. Gang, Valence-programmable nanoparticle architectures. *Nat. Comm.* **11**, 2279 (2020).

62. K. Günther, M. Mertig, R. Seidel, Mechanical and structural properties of YOYO-1 complexed DNA. *Nucleic Acids Res.* **38**, 6526–6532 (2010).

63. J. R. Kremer, D. N. Mastronarde, J. R. McIntosh, Computer visualization of three-dimensional image data using IMOD. *J. Struct. Biol.* **116**, 71–76 (1996).

64. J. Zivanov, T. Nakane, B. O. Forsberg, D. Kimanius, W. J. Hagen, E. Lindahl, S. H. Scheres, New tools for automated high-resolution cryo-EM structure determination in RELION-3. *eLife* **7**, e42166 (2018).

65. A. Rohou, N. Grigorieff, CTFFIND4: Fast and accurate defocus estimation from electron micrographs. *J. Struct. Biol.* **192**, 216–221 (2015).

66. J. SantaLucia Jr., D. Hicks, The thermodynamics of DNA structural motifs. *Annu. Rev. Biophys. Biomol. Struct.* **33**, 415–440 (2004).

67. R. Phillips, J. Kondev, J. Theriot, H. Garcia, *Physical Biology of the Cell* (Garland Science, 2012).
